# Deacetylase inhibitors repress STAT5-mediated transcription by interfering with bromodomain and extra-terminal (BET) protein function

**DOI:** 10.1093/nar/gkv188

**Published:** 2015-03-13

**Authors:** Sophia Pinz, Samy Unser, Dominik Buob, Philipp Fischer, Belinda Jobst, Anne Rascle

**Affiliations:** Stat5 Signaling Research Group, Institute of Immunology, University of Regensburg, 93053 Regensburg, Germany

## Abstract

Signal transducer and activator of transcription STAT5 is essential for the regulation of proliferation and survival genes. Its activity is tightly regulated through cytokine signaling and is often upregulated in cancer. We showed previously that the deacetylase inhibitor trichostatin A (TSA) inhibits STAT5-mediated transcription by preventing recruitment of the transcriptional machinery at a step following STAT5 binding to DNA. The mechanism and factors involved in this inhibition remain unknown. We now show that deacetylase inhibitors do not target STAT5 acetylation, as we initially hypothesized. Instead, they induce a rapid increase in global histone acetylation apparently resulting in the delocalization of the bromodomain and extra-terminal (BET) protein Brd2 and of the Brd2-associated factor TBP to hyperacetylated chromatin. Treatment with the BET inhibitor (+)-JQ1 inhibited expression of STAT5 target genes, supporting a role of BET proteins in the regulation of STAT5 activity. Accordingly, chromatin immunoprecipitation demonstrated that Brd2 is associated with the transcriptionally active STAT5 target gene *Cis* and is displaced upon TSA treatment. Our data therefore indicate that Brd2 is required for the proper recruitment of the transcriptional machinery at STAT5 target genes and that deacetylase inhibitors suppress STAT5-mediated transcription by interfering with Brd2 function.

## INTRODUCTION

Signal transducer and activator of transcription STAT5 is an essential regulator of cell differentiation, proliferation and survival (1–3). Following stimulation with specific cytokines, growth factors and hormones, the latent transcription factor is phosphorylated by receptor-associated JAK tyrosine kinases. STAT5 phosphorylation allows its dimerization and translocation into the nucleus where it binds to conserved recognition sites usually present in the proximal promoters of STAT5 target genes, resulting in their transcriptional activation (3–5). In normal cells, STAT5 activity is tightly controlled via attenuation mechanisms including dephosphorylation by SHP-1 phosphatase and a negative feedback loop by CIS/SOCS family proteins (6,7). STAT5 activity is further regulated by post-translational modifications (acetylation, SUMOylation) (2,8–11) and by protein interactions with itself (tetramerization), transcription factors, transcriptional coactivators and corepressors, and with chromatin modifying enzymes (2,12–18). STAT5 activity is frequently deregulated in cancer cells, which typically exhibit constitutively phosphorylated STAT5 due to the aberrant activity of oncogenic kinases or as a result of point mutations in STAT5 proteins (19–26). STAT5 constitutive activation results in increased cell proliferation and reduced cell apoptosis, and is as such an important player in cancer initiation and progression (3,6,27–30). The STAT5 pathway is therefore an acknowledged target for cancer prevention and therapy (31–33). A number of inhibitors of the STAT5 pathway have been described, including tyrosine kinase inhibitors targeting JAK family members and small-molecule inhibitors targeting STAT5 phosphorylation or DNA binding activity (34–39). Inhibitors targeting STAT5 transcriptional activity, at a step subsequent to its binding to DNA, have been described. They include deacetylase inhibitors such as trichostatin A (TSA), suberoylanilide hydroxamic acid (SAHA) and sodium butyrate (NaB), initially described by our group, and more recently the bromodomain inhibitor (+)-JQ1 and the natural compound sulforaphane (40–43). The mechanism of inhibition of STAT5 activity by these inhibitors remains unknown. Therapies combining tyrosine kinase inhibitors and either deacetylase or bromodomain inhibitors, as well as therapies combining deacetylase and bromodomain inhibitors proved to be more effective in the treatment of cancers with constitutive active STAT5 (42,44–49). A number of deacetylase inhibitors are either approved or currently being evaluated for the treatment of various types of cancers (50). Understanding the mode of action of these inhibitors is thus not only fundamental for a better characterization of their activity and specificity but will also contribute to a better understanding of the mechanism of transcriptional regulation by STAT5 in normal and cancer cells.

We showed before that the deacetylase inhibitors TSA, SAHA and NaB inhibit cytokine-induced STAT5 transcriptional activity by preventing recruitment of components of the transcriptional machinery (TBP, RNA polymerase II) without affecting STAT5 binding to DNA (40). Analysis of histone H3 and H4 acetylation revealed minor changes upon TSA treatment along the STAT5 target gene *Cis*, as opposed to the control gene *c-Fos* (41). Beside, histone acetylation levels remained particularly low in the vicinity of the STAT5 binding sites within the *Cis* promoter, regardless of interleukin-3 (IL-3) stimulation or TSA treatment (41). Therefore, our previous data suggested that deacetylase inhibitors are unlikely to modulate histone acetylation locally and rather block STAT5 activity by altering properties of the DNA-bound STAT5 protein. The present study aimed at identifying the molecular mechanism of inhibition of STAT5 activity by deacetylase inhibitors. STAT5 and other STAT proteins are known to be acetylated on specific lysines located within their C-terminal transactivation domain, DNA binding domain and/or N-terminal domain (9,10,51–56). The effect of acetylation and deacetylation on STAT activity varies with the respective STAT protein. The existence of contradictory reports also suggest complex spatio-temporal regulatory mechanisms that might also depend on the cytokine-activated receptors involved and the cell type investigated (9,11,13,53–55,57–61). STAT1 acetylation on K410 and K413 within its DNA binding domain inhibits its phosphorylation and thus its transcriptional activity, in agreement with the requirement of a deacetylase (HDAC) activity for STAT1 signaling (51,58,62). By contrast STAT2 acetylation on K390 (DNA binding domain) is required for the transcriptional activity of the STAT1/STAT2/IRF9 (ISGF3) complex induced by IFNα (52). A number of conflicting results have been reported regarding the identity of acetylated lysine(s) within STAT3 and their implication in regulating STAT3 activity. STAT3 acetylation in the cytosol on a single lysine (K685) within the C-terminal SH2 domain did not influence STAT3 phosphorylation and nuclear translocation but its dimerization, thus eventually modulating its DNA binding and transcriptional activities (53,60). By contrast, Nie *et al*. showed that acetylation of K685 together with K679, K707 and K709 is required for STAT3 phosphorylation and downstream activity (54). This group also showed that acetylation of N-terminal lysines K49 and K87 does not play a role in STAT3 activity, which contradicts another report proposing that acetylation at K49 and K87 takes place at a step following STAT3 phosphorylation, nuclear translocation and binding to DNA, to allow recruitment and stabilization of p300 and RNA polymerase II, and thus transcription (55,61). Cytosolic STAT5B acetylation in response to prolactin at K359 (DNA binding domain) and K694 (K689 in STAT5A) and to a lesser extent at K701 (K696 in STAT5A), both within the C-terminal SH2 domain, did not influence STAT5 phosphorylation but its dimerization, thus affecting its activity (10). STAT5A acetylation at K696, but not at K700, was also reported independently (8,56). Interestingly, STAT5 is also SUMOylated at the same K696 residue. Mutating K696 to arginine partially affected growth hormone (GH)-induced STAT5 activity in a luciferase reporter assay in mouse embryonic fibroblasts (8), suggesting that acetylation and/or SUMOylation at K696 might play a role in the regulation of STAT5 activity.

Existing data thus suggest a possible role of STAT5 acetylation at K359, K689 and/or K696 independent of STAT5 phosphorylation (8–11,56,57). We then hypothesized that the observed inhibitory effect of deacetylase inhibitors on STAT5 transcriptional activity might involve acetylation of STAT5, possibly resulting in the disruption of protein–protein interaction(s) between STAT5 and essential components of the transcriptional machinery. Therefore our study initially aimed at (i) identifying essential lysine residues within STAT5 that are potentially acetylated upon treatment with deacetylase inhibitors such as TSA, (ii) correlating STAT5 acetylation with a possible alteration in the composition of STAT5-containing complexes upon TSA treatment and (iii) identifying among HDAC1–11 (63,64) the deacetylase(s) responsible for this effect. We found that mutating multiple lysine residues within constitutively active STAT5A-1*6 did not affect its transcriptional activity in Ba/F3 cells. Besides, TSA treatment did not affect the gel filtration profile of STAT5-containing complexes. These observations strongly argued against a direct inhibition of STAT5 activity through alteration of STAT5 acetylation. Interestingly, the ability of the deacetylase inhibitors TSA, valproic acid (VPA) and apicidin to inhibit STAT5 activity correlated with a rapid and strong increase in global histone acetylation. By contrast, neither STAT5 activity nor global histone acetylation was affected by the deacetylase inhibitors MGCD0103 and MS-275. The global increase in histone acetylation induced by TSA treatment was associated with local changes in histone acetylation and a decrease in histone occupancy at several gene loci, likely reflecting nucleosome instability provoked by increased acetylation. We therefore investigated whether changes in chromatin structure might interfere with the function of chromatin-associated factors, in particular of those interacting with acetylated histones. We found that upon treatment with TSA, VPA and apicidin—but not with MGCD0103 and MS-275—the bromodomain and extra-terminal (BET) nuclear protein Brd2, and to a lesser extent the TATA-box binding protein TBP, were rapidly depleted from the soluble nuclear fraction but not from the insoluble chromatin fraction. This finding suggests a rapid delocalization of Brd2 and of the Brd2-associated factor TBP to hyperacetylated chromatin. Further experiments using the bromodomain inhibitor (+)-JQ1 and chromatin immunoprecipitation (ChIP) assays using Brd2 antibodies further confirmed a role of Brd2 in regulating STAT5 activity. Altogether, our data demonstrate, to our knowledge for the first time, that deacetylase inhibitors block STAT5 transcriptional activity by interfering with the function of the BET family protein Brd2, very likely preventing it from recruiting and stabilizing the transcriptional machinery. Our findings represent a major step not only in understanding the mechanism of transcriptional regulation by the oncoprotein STAT5 but also in characterizing the mode of action and specificity of deacetylase inhibitors.

## MATERIALS AND METHODS

### Chemicals

Dimethyl sulfoxide (DMSO), TSA, MG132 and insulin were purchased from SIGMA (D-2650, T-8552, C-2211 and I-9278, respectively). (+)-JQ1 (BPS Bioscience #27401)—hereafter abbreviated to JQ1—was purchased from BIOMOL GmbH. VPA, apicidin and MS-275 were purchased from Enzo Life Sciences (ALX-550–304, BML-GR340 and ALX-270–378, respectively). MGCD0103 (Selleck S1122) was purchased from Absource Diagnostics GmbH. With the exception of VPA, which was dissolved at 300 mM in H_2_O, compounds were dissolved in DMSO at a final concentration of 1 mM (TSA), 10 mM (Apicidin), 40 mM (MGCD0103), 25 mM (MS-275), 5 mM (JQ1) and 10 mM (MG132). DMSO was used as vehicle control. Its final concentration was adjusted in all conditions, unless indicated otherwise, and never exceeded 0.12%.

### Antibodies

Antibodies used for western blot and ChIP were pSTAT5 (Cell Signaling Technology 9351), STAT5A (Santa Cruz Biotechnology sc-1081), STAT5B (Santa Cruz Biotechnology sc-1656), STAT5A+B (Santa Cruz Biotechnology sc-835), RNA polymerase II (Santa Cruz Biotechnology sc-899 and sc-900), TBP (Santa Cruz Biotechnology sc-273), histone H3 (Abcam ab1791), acetylated histone H3 (Ac-H3; Millipore 06–599), acetylated histone H4 (Ac-H4; Millipore 06–866), α-tubulin (Santa Cruz Biotechnology sc-32293), HDAC1 (Millipore 05–100), HDAC2 (Invitrogen 51–5100), HDAC3 (Cell Signaling Technology 2632), FLAG (M2, SIGMA F-1804), Brd2 (Bethyl A302–583A) and IgG from rabbit serum (SIGMA I-5006; isotype control for ChIP). Secondary antibodies for western blot were anti-Rabbit IgG-Peroxidase (SIGMA A-0545) and anti-Mouse IgG-Peroxidase (SIGMA A-8924).

### Cell lines and drug treatments

All cell lines were cultivated at 37°C under 5% CO_2_ in a humidified incubator. The IL-3-dependent mouse pro-B cell line Ba/F3 (a kind gift from Jacqueline Marvel, IFR 128 BioSciences Gerland-Lyon Sud, France) (65) was grown in RPMI 1640 (PAN-Biotech P04–16500) supplemented with 10% heat-inactivated fetal calf serum (FCS; PAN-Biotech), 1% penicillin/streptomycin (PAN-Biotech) and 2 ng/ml rmIL-3 (ImmunoTools). The IL-3-independent Ba/F3–1*6 cell line (clone F7) stably expressing the constitutively active (FLAG-tagged) mouse STAT5A-1*6 mutant (66) has been recently described (36) and was grown in RPMI 1640 supplemented with 10% heat-inactivated FCS, 1% penicillin/streptomycin and 600 μg/ml G418 (SIGMA A-1720). The Ba/F3-tet-on-1*6 cell line was generated by stably transfecting Ba/F3 cells with the pTet-On Advanced vector (Clontech) expressing the rtTA-Advanced transactivator under neomycin (G418) selection. A clone highly expressing the transactivator was selected and stably co-transfected with a tetracycline-responsive vector conditionally expressing FLAG-tagged mouse STAT5A-1*6 (pTRE-Tight-BI-AcGFP1-mSTAT5A-1*6-FLAG) and pTK-Hyg (Clontech) to confer hygromycin B resistance. Upon hygromycin B selection, clones highly expressing mSTAT5A-1*6-FLAG in the presence of 1 μg/ml of the tetracycline analog doxycycline (SIGMA D-9891) were selected and further characterized (paper in preparation). Ba/F3-tet-on-1*6 clone D4.1 was chosen for our study. Non-induced Ba/F3-tet-on-1*6 cells were grown in RPMI 1640 supplemented with 10% heat-inactivated FCS, 1% penicillin/streptomycin, 600 μg/ml G418, 800 μg/ml hygromycin B (PAN-Biotech) and 2 ng/ml rmIL-3. Expression of mSTAT5A-1*6-FLAG was induced by growing the cells 24 h in RPMI 1640 supplemented with 10% heat-inactivated FCS, 1% penicillin/streptomycin and 1 μg/ml doxycycline.

For cytokine stimulation of Ba/F3 cells, cells were washed twice in RPMI 1640 and rested in RPMI 1640/10% FCS/1% penicillin-streptomycin for 6–12 h before addition of 2–5 ng/ml IL-3 for 30–120 min, as indicated.

For inhibitor treatment of Ba/F3 cells, unless indicated otherwise, rested cells were pre-treated 30 min with the respective compound or with DMSO (vehicle) prior to IL-3 stimulation. Ba/F3–1*6 cells were treated with inhibitors or vehicle for 5–240 min, as indicated.

### Plasmid transfection by electroporation

Lysine to glutamine (K>Q) and lysine to arginine (K>R) point mutations were introduced within mouse STAT5A-1*6 (K84, K359, K384, K675, K681, K689, K696, K700) of pcDNA3-mSTAT5A-1*6-FLAG vector (36) by following the QuikChange site-directed mutagenesis protocol (oligonucleotides used shown in Supplementary Table S1). Multiple mutants (the so-called 2xQ/R for the K696/K700 double K>Q or K>R mutants, 3xQ/R for the K689/K696/K700 triple K>Q or K>R mutants and 5xQ/R for the K675/K681/K689/K696/K700 quintuple K>Q or K>R mutants) were also generated. As a positive control for inactivating mutation, the tyrosine residue at position 694 of mSTAT5A-1*6, which is essential for its phosphorylation and activity (66), was mutated to phenylalanine (Y694F). All mutations were confirmed by sequencing. Plasmid pcDNA3-mSTAT5A-FLAG, expressing wild-type mouse STAT5A, was used as a negative control for STAT5 activity in IL-3-free medium. Empty pcDNA3 vector (Invitrogen) was also tested in parallel.

Expression plasmids were prepared with the Plasmid Pure Midi Kit (Qiagen) and transfected into Ba/F3 cells by electroporation using the Gene Pulser Xcell Electroporation System with CE Module (Bio-Rad). Briefly, 4 × 10^6^ cells were mixed with 8 μg plasmid DNA in 800 μl RPMI 1640, transferred into a 0.4 cm gap cuvette (VWR) and electroporated by delivering one exponential decay pulse of 320 V and 950 μF. Transfected cells were returned to RPMI 1640/10% FCS/1% penicillin–streptomycin for 10 h in the absence of IL-3, and harvested for gene expression and western blot analyses, as described below.

### siRNA transfection by electroporation

HDAC-specific and ScI control siRNAs (Supplementary Table S1) were transfected into Ba/F3 and Ba/F3–1*6 cells by electroporation using either the Bio-Rad Gene Pulser II with RF Module (Supplementary Figure S4 A–D) as previously described (41), or the Bio-Rad Gene Pulser Xcell with CE Module (Supplementary Figure S4 E–G) as follows. Ba/F3–1*6 cells were subjected to two rounds of electroporation (at *t* = 0 and at 24 h) and harvested at 48–72 h for gene expression and western blot analyses. Per transfection, PBS-washed 1 × 10^6^ cells were resuspended in 100 μl Gene Pulser Electroporation Buffer (Bio-Rad) containing 0.5 to 1 μM siRNA duplexes, transferred to a 0.1 cm gap cuvette (VWR) and electroporated through delivery of two square wave pulses of 95V, 5 ms duration and 0.1 s interval. Transfected cells were returned to RPMI 1640 supplemented with 10% FCS and 1% penicillin/streptomycin. Multiple siRNA transfections were performed using equivalent amounts of each siRNA up to the final siRNA concentration indicated.

### Gene expression analysis by quantitative RT-PCR

Following inhibitor and cytokine treatments, cells were harvested, lysed in the iScript RT-qPCR sample preparation reagent (170–8899, Bio-Rad Laboratories) at a concentration of 4 × 10^5^ cells/ml and 1 μl was used for cDNA synthesis using the iScript cDNA Synthesis kit (170–8891, Bio-Rad Laboratories), following the manufacturer's instructions. Quantitative PCR was performed on a RotorGene Q (Qiagen) using a 2-step PCR program (95°C 15 s, 60°C 60 s; 40 cycles). Twenty microliter quantitative PCR reactions were performed using 0.4 μl cDNA template and a self-made master-mix containing SYBR Green I and HotStarTaq (Qiagen). Mouse-specific quantitative PCR primers used are listed in Supplementary Table S1. Data were normalized to mouse S9 or 36b4 ribosomal mRNAs and expressed as relative mRNA levels, like previously reported (4,36,40,41,43,67). Data are mean ± SD of the quantitative PCR performed in either duplicate or triplicate and are representative of at least two independent experiments.

### Quantitative chromatin immunoprecipitation (ChIP) assays

ChIP of STAT5, RNA polymerase II, total histone H3, acetylated histones H3/H4 and Brd2 (Ba/F3 cells) was performed from whole cell extracts as previously described (36,40,43). Improved Brd2 detection in Ba/F3–1*6 cells by ChIP was achieved using an alternative protocol of nuclei fractionation and chromatin shearing, adapted from Métivier *et al*. (68) and Okada and Fukagawa (69) respectively, with the following modifications. Formaldehyde-crosslinked cells (36,40,43) were sequentially lysed following Métivier's protocol (68). Nuclei were resuspended in HDG150 buffer (20 mM HEPES pH 7.6, 150 mM KCl, 10% glycerol, 0.5 mM DTT, 10 mM NaF, 10 μg/ml leupeptin, 10 μg/ml aprotinin, 0.5 mM phenylmethylsulfonyl fluoride) and sonicated six times for 20 s on a Branson Sonifier 250 (output 3, 50% duty cycle). Chromatin was further fragmented by MNase digest (SIGMA N-3755; reconstituted in 5 mM Tris pH 6.8, 50 mM NaCl, 50% glycerol) using 0.25 U MNase/10^6^ cells for 60 min at +4°C in the presence of 3 mM CaCl_2_. MNase activity was stopped with 5 mM EGTA and the sheared nuclear lysate was diluted 5-fold in immunoprecipitation (IP) buffer (40). After centrifugation (3000 x *g*, 10 min at +4°C), the supernatant was further precleared against protein A-sepharose and ChIP conducted as previously described (36,40,43), using 3 μg of Brd2-specific antibody or of rabbit IgG as a control.

Co-precipitated genomic DNA was quantified by quantitative PCR using primers listed in Supplementary Table S1. Data are expressed as percentage (%) of input DNA or—when histone H3 and H4 acetylation was normalized to histone H3 content—as Ac-H3/H3 and Ac-H4/H3 % of input DNA ratios. Data are mean ± SD of the quantitative PCR performed in either duplicate or triplicate and are representative of at least two independent experiments.

### Protein analysis

For Brij whole-cell protein lysis, cells were resuspended in Brij Lysis buffer (10 mM Tris-HCl pH 7.5, 150 mM NaCl, 2 mM EDTA pH 8.0, 0.875% Brij 97, 0.125% NP40, 10 mM NaF, 1 mM Na_3_VO_4_, 10 μg/ml leupeptin, 10 μg/ml aprotinin, 0.5 mM phenylmethylsulfonyl fluoride), lysed by incubation 30 min at +4°C with occasional stirring, and debris was eliminated by centrifugation for 15 min at 20,000 x *g* at +4°C.

Freeze-thaw whole-cell protein lysis for the analysis of histone protein level and acetylation was performed as previously described (43).

For cytosolic and nuclear protein lysis, cells were gently resuspended in buffer A (10 mM HEPES pH 7.6, 15 mM KCl, 2 mM MgCl_2_, 0.1 mM EDTA, 10 mM NaF, 10 μg/ml leupeptin, 10 μg/ml aprotinin, 0.5 mM phenylmethylsulfonyl fluoride) containing 0.1–0.2% NP40, centrifuged at 500 x *g* for 1 min and the supernatant collected (cytosolic fraction). Nuclei were washed once in buffer A and lysed by incubation 30 min at +4°C in buffer C (50 mM HEPES pH 7.9, 400 mM KCl, 0.1 mM EDTA, 10% glycerol, 10mM NaF, 10 μg/ml leupeptin, 10 μg/ml aprotinin, 0.5 mM phenylmethylsulfonyl fluoride). Insoluble particles were eliminated by centrifugation (15 min at 20,000 x *g*) and the cleared supernatant (nuclear fraction) was collected.

For subfractionation of nuclei into soluble and insoluble fractions, cells were first lysed in buffer A containing 0.1% NP40 (cytosolic extraction), and nuclei washed once in buffer A, as above. Nuclei were resuspended in Brij Lysis buffer and incubated for 30 min at +4°C on a rotating wheel. After centrifugation at 1000 x *g*, the supernatant containing the soluble nuclear proteins was collected. The insoluble nuclear pellet was resuspended in the same volume of Brij Lysis buffer, freezed-thawed once and treated with 0.1 mg/ml DNase I in the presence of 7 mM MgCl_2_ for 30 minutes at +4°C on wheel. One volume of 2x Laemmli buffer containing β-mercaptoethanol was added, samples were heated at 95°C for 10 minutes while vortexed regularly. Denatured samples were centrifuged 10 minutes at maximum speed at room temperature, to eliminate remaining insoluble debris, and the supernatant corresponding mainly to the chromatin fraction was collected. It should be noted that a small viscous pellet remained at this last centrifugation step, indicating that a certain amount of undigested DNA and possibly associated proteins were not extracted.

Immunoblotting was performed as recently described (36).

### Gel filtration

For gel filtration chromatography and analysis of STAT5-containing protein complexes, nuclear lysates from 5 × 10^7^ Ba/F3–1*6 cells treated for 60 min with 200 nM TSA or 0.02% DMSO (vehicle) were prepared in buffer C as described above, dialyzed 50 min against GF buffer (25 mM HEPES pH 7.2, 0.1 M KCl, 0.1 mM EDTA, 5% glycerol, 1 mM DTT, 100 μg/ml insulin, 1 mM phenylmethylsulfonyl fluoride, 2 mM benzamidine-HCl) and debris was removed by centrifugation. TSA-treated samples were processed in buffers containing 20 nM TSA. Sample volume was adjusted to 600 μl and 500 μl was loaded on a TSK-Gel 4000SW column (Tosoh Bioscience) and separated using ÄKTA FPLC system (GE Healthcare). Thyroglobulin (669 kDa) and BSA (66 kDa) were used as protein standards. Proteins were eluted in GF buffer at a flow rate of 0.5 ml/min. Fractions of 500 μl volume were collected and analysed by western blot using antibodies directed against FLAG tag (recognizing mSTAT5A-1*6-FLAG fusion protein), STAT5A+B and RNA polymerase II.

## RESULTS

### STAT5 acetylation at multiple lysines is not essential for its transcriptional activity in Ba/F3 cells

**Figure 1.**
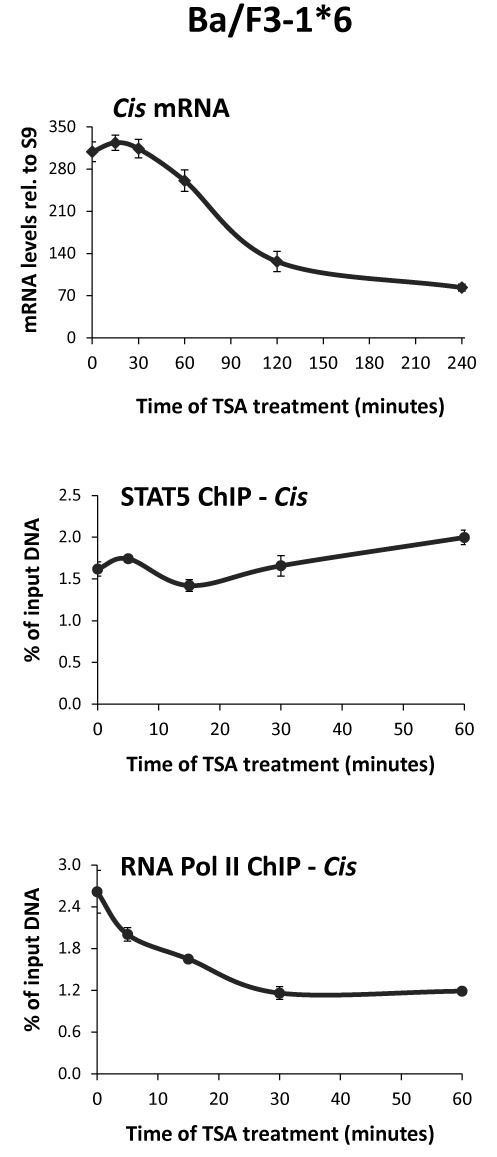
Constitutive STAT5 activity is inhibited by the deacetylase inhibitor TSA. Ba/F3–1*6 cells expressing mouse constitutive active STAT5A-1*6 mutant were treated with 200 nM TSA and harvested at the indicated times. Expression of the STAT5 target gene *Cis* was investigated by quantitative RT-PCR. Binding of STAT5A-1*6 to the *Cis* promoter and recruitment of RNA polymerase II to the transcription start site of the *Cis* gene were monitored by ChIP using primers Cis -188/-104 and -18/+55, respectively (Supplementary Table S1).

We showed before that deacetylase inhibitors such as TSA, SAHA or NaB inhibit STAT5 transcriptional activity by preventing recruitment of TBP and RNA polymerase II to the promoter of target genes such as *Cis* and *Osm*, at a step following STAT5 binding to DNA (40). STAT proteins, in particular STAT3 and STAT5, are known to be regulated by acetylation of multiple lysines (8–10,53–56). Therefore, we investigated whether alteration of STAT5 acetylation might explain the observed effect of deacetylase inhibitors on STAT5 activity by introducing mutations at specific lysine residues. To prohibit a possible interference with STAT5 initial activation (phosphorylation, dimerization, nuclear translocation, DNA binding) and instead directly address an effect on STAT5 transcriptional activity, mutations were introduced in the constitutively active STAT5A-1*6 protein (66). So far, inhibition of STAT5 transcriptional activity by deacetylase inhibitors was only demonstrated in cells expressing wild-type STAT5 (40,41). Therefore, we first verified that constitutive activity of STAT5A-1*6 was equally sensitive to TSA. Upon treatment of IL-3-independent Ba/F3–1*6 cells with 200 nM TSA, constitutive expression of STAT5 target genes such as *Cis* was downregulated (Figure 1 and data not shown). ChIP assays confirmed that RNA polymerase II occupancy at the *Cis* transcription start site was reduced in a time-dependent manner, in correlation with the transcriptional inhibition, while STAT5 DNA binding activity remained unaffected (Figure 1). Thus, in line with our reported observations on wild-type STAT5 in Ba/F3 cells (40,41), transcription mediated by constitutively active STAT5A-1*6 is also impaired upon treatment with deacetylase inhibitors, likely through destabilization of the transcriptional machinery.

**Figure 2.**
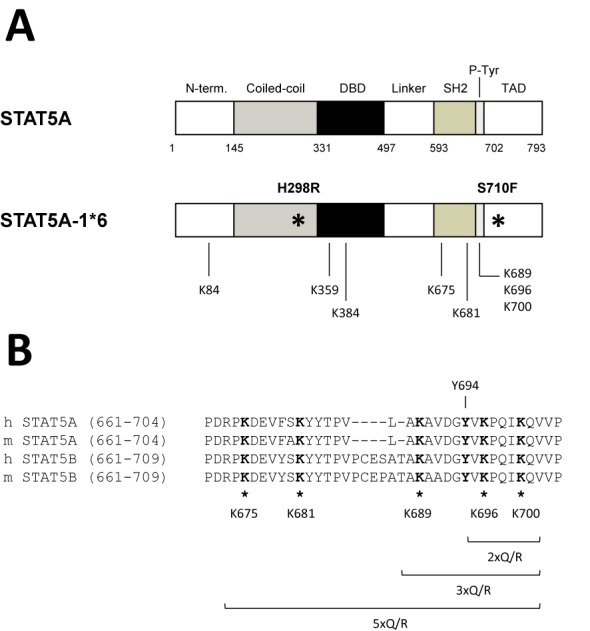
Structure of the STAT5A-1*6 lysine mutants. (**A**) Schematic representation of mouse wild-type and constitutively active (1*6) STAT5A proteins. STAT5A-1*6 presents two point mutations (H298R, S710F; *) responsible for its constitutive activity (66). STAT5A and STAT5A-1*6 cDNAs were subcloned into the pcDNA3 expression vector, in frame with a C-terminal FLAG tag (not shown), to allow protein detection by western blot. Specific lysine residues (K84, K359, K384, K675, K681, K689, K696, K700) were mutated to glutamine (Q) or to arginine (R) by site-directed mutagenesis. (**B**) Sequence alignment of the C-terminal SH2 and transactivation domains of mouse (m) and human (h) STAT5A and STAT5B highlighting conserved lysine residues (*). Lysine residues mutated within mouse STAT5A-1*6 C-terminal domain are specified (K675, K681, K689, K696, K700). Double (2xQ/R), triple (3xQ/R) and quintuple (5xQ/R) STAT5A-1*6 lysine mutants were also generated, as indicated. The essential and constitutively phosphorylated tyrosine Y694 of mouse STAT5A-1*6 was also mutated into phenylalanine (Y694F), as a control for STAT5A-1*6 inactivation. N-term., N-terminal domain; DBD, DNA binding domain; P-Tyr, phospho-tyrosine tail; TAD, transactivation domain.

**Figure 3.**
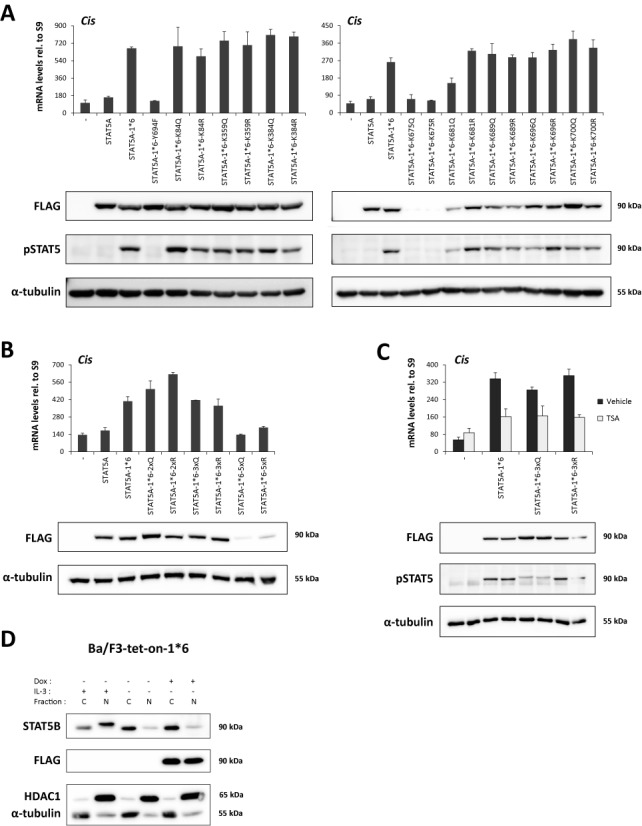
Lysine acetylation is not required for STAT5A-1*6 transcriptional activity in Ba/F3 cells. (**A–C**) Ba/F3 cells were transfected with empty pcDNA3 (-) or pcDNA3-based plasmids expressing wild-type mSTAT5A, constitutively active mSTAT5A-1*6 or mSTAT5A-1*6 mutants (K>Q, K>R and Y694F), as indicated. Transfected cells were maintained for 10 h in IL-3-free medium to prevent activation of endogenous STAT5. In (C), transfected cells were treated with 200 nM TSA or 0.02% DMSO (vehicle) for the last 90 min before harvest. Transgene expression and phosphorylation of STAT5 proteins were verified by western blot analysis of Brij whole-cell lysates using FLAG- and pSTAT5-specific antibodies, respectively. α-Tubulin was used as a loading control. Expression of the STAT5 target gene *Cis* was investigated by quantitative RT-PCR, as before. Of note, data not shown suggest that the weaker pSTAT5 signal consistently detected for the 3xQ mutant is likely due to epitope masking, probably as a result of multiple adjacent K>Q mutations surrounding the phospho-tyrosine residue. (**D**) Ba/F3-tet-on-1*6 cells were grown for 22 h in the absence or presence of 1 μg/ml doxycycline (Dox) to induce mSTAT5A-1*6 expression. Cells were kept in IL-3-containing medium for the first 12 h, washed twice in RPMI 1640 and further cultivated in IL-3-deprived medium for 10 h (±Dox, -IL-3) until harvest to inactivate endogenous STAT5 activity. As a positive control for endogenous STAT5 activity, non-induced cells were maintained in IL-3-containing medium (-Dox, +IL-3) for the duration of the experiment (22 h). Cytosolic and nuclear protein lysates were analysed by western blot to monitor endogenous nuclear STAT5B protein level (STAT5B) before and after induction of STAT5A-1*6 (FLAG). α-Tubulin and HDAC1 served as cytosolic and nuclear markers, respectively, to verify the quality of cell fractionation.

Lysine to glutamine (K>Q) and lysine to arginine (K>R) substitutions that mimic the acetylated or unmodified lysine respectively (70) were then introduced into STAT5A-1*6. Lysine residues that were proposed to be critical for the regulation of STAT5 but also of STAT3 at equivalent positions (8–10,53–56,60)—namely K84, K359, K384, K675, K681, K689, K696 and K700—were mutated either individually or in combination (Figure 2). Transcriptional activity of the STAT5A-1*6 lysine mutants was assessed upon transfection in Ba/F3 cells maintained in IL-3-free medium, hence in conditions where endogenous wild-type STAT5 is inactive. Expression (protein level) and activation (phosphorylation) of the respective STAT5A-1*6 mutants were verified by western blot, and their transcriptional activity was evaluated by measuring expression of the STAT5 target gene *Cis* by quantitative RT-PCR. Neither single nor multiple lysine mutations impaired STAT5A-1*6 transcriptional activity (Figure 3A and B). Of note, STAT5A-1*6 proteins mutated at lysine 675 (K675Q, K675R as well as 5xQ and 5xR) were poorly detectable in western blot, likely due to protein instability, and thus their activity could not be readily assessed. Nevertheless, these results indicate that acetylation at the investigated lysines, notably at K359, K689 or K696 previously reported to be acetylated and important for STAT5 activity (8,10,56), is not required for STAT5A-1*6 activity in Ba/F3 cells. These experiments therefore indicate that the impaired STAT5 transcriptional activity induced by deacetylase inhibitors is unlikely to be due to a change in STAT5 acetylation, at least at the investigated lysines. This proposition is further supported by the important finding that the transcriptional activity of STAT5A-1*6 3xQ/R proteins, mutated simultaneously at K689, K696 and K700, remained sensitive to TSA (Figure 3C). Of note, the observation that the STAT5A-1*6 K696Q/R mutants are as active as the non-mutated STAT5A-1*6 protein indicates that not only acetylation but also SUMOylation at K696 is not required for STAT5A-1*6 transcriptional activity in Ba/F3 cells.

To rule out that the transcriptional activity of the STAT5A-1*6 lysine mutants was mediated by the co-translocation of endogenous wild-type STAT5 proteins to the nucleus—despite the absence of IL-3—we used a Ba/F3-tet-on cell line which allows the conditional expression of STAT5A-1*6 in the presence of doxycycline (Figure 3D). The comparison of the level of endogenous nuclear STAT5B proteins before and after induction of STAT5A-1*6 showed no increase of endogenous STAT5 in the nucleus when STAT5A-1*6 was expressed (Figure 3D). In addition, no protein interaction between STAT5A-1*6 and latent endogenous STAT5B could be detected in co-immunoprecipitation assays (data not shown). It is therefore unlikely that the transcriptional activity of our STAT5A-1*6 mutants is mediated by endogenous STAT5 proteins. Overall, our data strongly argue against an implication of STAT5 acetylation in the mechanism of inhibition of STAT5-induced transcription by deacetylase inhibitors. On the other hand, we cannot fully exclude that an essential not-yet-described lysine within STAT5 may account for the observed effect.

Our initial hypothesis assumed that alteration of STAT5 acetylation might disrupt protein–protein interactions between STAT5 and the transcriptional machinery. We therefore compared the profiles of STAT5- and RNA polymerase II-containing complexes in nuclear extracts from Ba/F3–1*6 cells treated for 1 h with 200 nM TSA or DMSO (vehicle), which were produced by gel filtration chromatography (Supplementary Figure S1). No apparent alterations in the elution profiles were noticed upon treatment with the deacetylase inhibitor.

Taken together, these experiments indicate that TSA-mediated inhibition of STAT5 target gene expression occurs independently of STAT5 acetylation and is not associated with an apparent disruption of STAT5-containing protein complexes.

### STAT5 target gene expression is differentially affected by class I-selective deacetylase inhibitors

**Figure 4.**
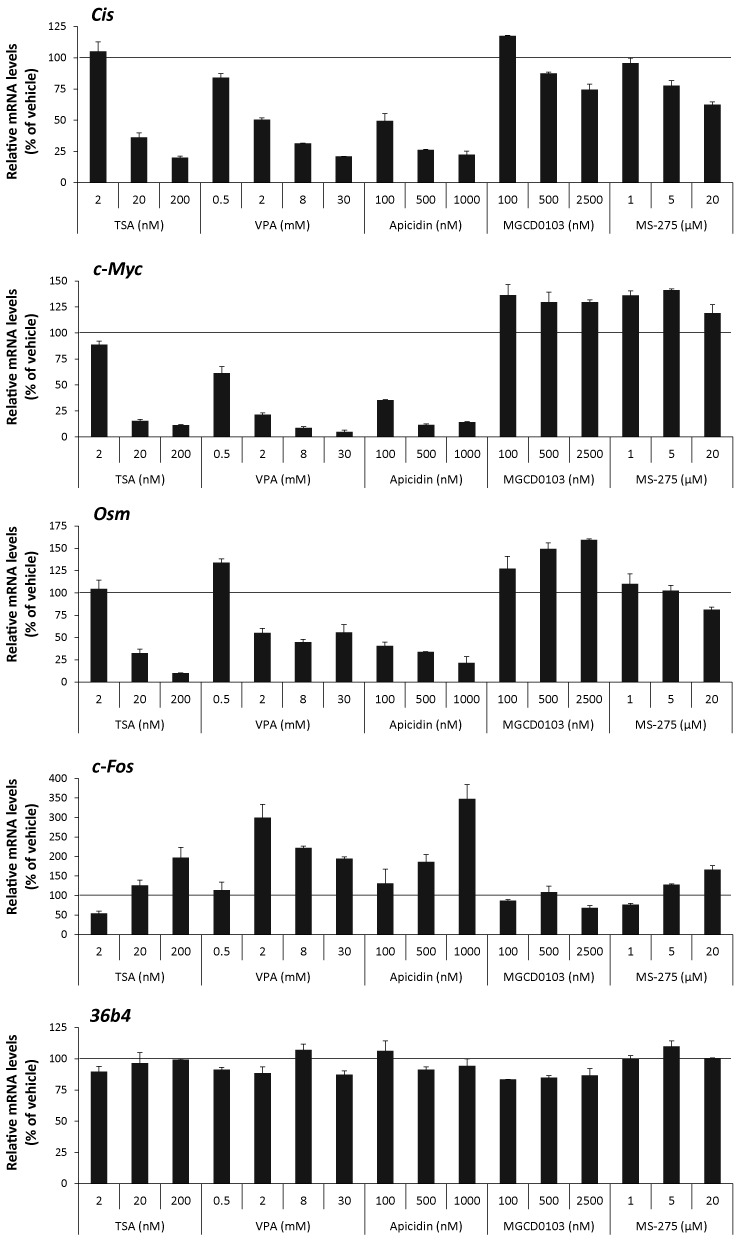
STAT5 activity in Ba/F3 cells is differentially affected by class I-selective deacetylase inhibitors. Rested Ba/F3 cells were pre-treated with the indicated concentrations of the pan-inhibitor TSA and of the class I-selective deacetylase inhibitors VPA, apicidin, MGCD0103 and MS-275 for 15 min (TSA) or 30 min (all others), and further stimulated with IL-3 for 60 min, as described in Materials and Methods section. Final DMSO concentration was adjusted as detailed in legend to Supplementary Figure S2. Expression of STAT5 target (*Cis, c-Myc, Osm*) and control (*c-Fos, 36b4*) genes was evaluated by quantitative RT-PCR as before. Data are expressed as mRNA levels in IL-3-stimulated cells relative to vehicle control. Non-normalized data are shown in Supplementary Figure S2.

We next aimed to identify which of the 11 deacetylases (HDAC1–11) described so far (63,64) might be implicated in the effect mediated by deacetylase inhibitors. First, class I-selective deacetylase inhibitors were tested for their ability to inhibit STAT5 target gene expression. Then, the consequences of siRNA-mediated knockdown of putative HDAC candidates on STAT5 target gene expression were investigated. Ba/F3 cells were pre-treated 30 minutes with increasing amounts of VPA, apicidin, MGCD0103 and MS-275 before stimulation with IL-3 for 1 h. Expression of STAT5 target (*Cis, c-Myc, Osm*) and control (*c-Fos, 36b4*) genes (4,36,40,41,43) was measured by quantitative RT-PCR (Figure 4 and Supplementary Figure S2). Similarly to TSA (Figure 4 and (40)), VPA and apicidin specifically inhibited STAT5-mediated transcription of *Cis, c-Myc* and *Osm*, while upregulating expression of the control gene *c-Fos*. By contrast, treatment with MGCD0103 and MS-275 had either marginal or no effects on the expression of all genes investigated. Expression of the Housekeeping gene *36b4* remained unaffected in all conditions (Figure 4). The deacetylase inhibitor concentrations used were in the range of the IC_50_ reported in the literature for the *in vitro* inhibition of recombinant human HDACs (Supplementary Table S2), and therefore were only indicative of their potential activity in cell culture. Nevertheless, the comparison of the concentration of deacetylase inhibitor necessary to inhibit STAT5 activity in Ba/F3 cells to the reported IC_50_ (Supplementary Table S2) supported the possibility that HDAC5, 7, 9 and 10 might represent potential candidate deacetylases for the regulation of STAT5 activity. Before knocking down the expression of *HDAC* genes, expression levels of individual HDACs in Ba/F3 cells were characterized by quantitative RT-PCR. While HDAC9 and HDAC11 mRNA levels were below the limit of detection, HDAC1–8 and HDAC10 were expressed at various levels in Ba/F3 cells, regardless of the presence of IL-3 (Supplementary Figure S3). Thus, the potential candidate HDAC9 is unlikely to be involved in TSA-mediated inhibition of STAT5 activity. Together, this preliminary screen suggested a possible implication of HDAC5, HDAC7 and/or HDAC10.

siRNA-mediated knockdown assays were then conducted to identify the HDAC(s) implicated in the regulation of STAT5-mediated transcription. Multiple experiments were conducted both in IL-3-stimulated Ba/F3 cells and in Ba/F3–1*6 cells, targeting not only the candidate HDACs mentioned above but also all 11 HDACs, individually as well as in a number of combinations (Supplementary Figure S4). The knockdown efficiency was verified at the RNA levels and, when western-blot-grade antibodies were available, at the protein level. Unexpectedly, none of the HDAC knockdowns performed had an effect on the expression of STAT5 target genes (Supplementary Figure S4). The observation that transfection of HDAC-specific siRNAs did not mimic the inhibitory effect of deacetylase inhibitors suggests that the knockdown efficiency was not sufficient or/and that several HDACs are functionally redundant. In support to these propositions, the level of histone acetylation in cells transfected with HDAC1-, 2-, 3- or 5-specific siRNAs was only marginally increased (Supplementary Figure S4F). On the other hand, transfection of HDAC expression plasmids had no effect on STAT5 target gene expression and histone acetylation (data not shown). In summary, the initial screen using class I-selective deacetylase inhibitors suggested a subset of HDACs as candidates possibly involved in STAT5 target gene expression. However, neither downregulation nor overexpression of the respective HDACs allowed a definite identification. Consequently, we decided to find alternative explanations for the observation that deacetylase inhibitors differentially affect STAT5 target gene expression.

### Histone acetylation is strongly and rapidly increased upon treatment with deacetylase inhibitors that inhibit STAT5-mediated transcription

Since STAT5 acetylation did not appear to account for the observed inhibition of STAT5-mediated transcription by the deacetylase inhibitors TSA, VPA and apicidin, we reassessed the possibility that changes in histone acetylation might be instead involved. We showed before that histone H3 and H4 acetylation along the *Cis* gene was at its lowest level in the vicinity of the STAT5 binding sites, which are located in the proximal promoter (41). Histone acetylation remained low at the STAT5 binding sites following both IL-3 stimulation and—most importantly—treatment with TSA (41). Moreover, TSA did not markedly alter the level of histone H3 and H4 acetylation at the promoter and within the open reading frame (ORF) of the *Cis* gene during IL-3 stimulation (41). Consequently, we had proposed that inhibition of STAT5 target gene expression by deacetylase inhibitors do not incriminate local alterations in histone acetylation (40,41). However, it remains possible that major alterations in chromatin structure at other loci might have an indirect impact on the regulation of STAT5 target genes. One drawback of our initial studies was that ChIP results from the analysis of histone acetylation at the *Cis* gene locus could not be corrected for changes in histone occupancy, since ChIP-grade and validated histone H3 antibodies were not available at that time. We therefore decided to reconsider the possibility that altered histone acetylation is involved in the inhibition of STAT5 target gene transcription upon treatment with deacetylase inhibitors.

**Figure 5.**
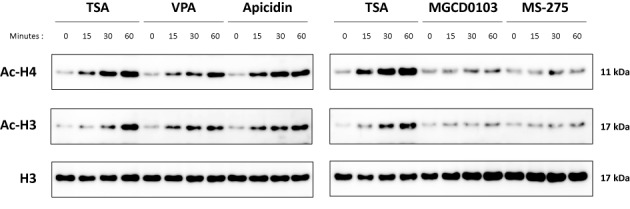
Histone acetylation is rapidly and specifically increased upon treatment with deacetylase inhibitors that inhibit STAT5 activity. Ba/F3 cells were treated for 0–60 min with 200 nM TSA, 3 mM VPA, 500 nM apicidin, 1 μM MGCD0103 or 5 μM MS-275. Freeze-thaw protein lysates were analysed by western blot using antibodies directed against acetylated histone H3 (Ac-H3), acetylated histone H4 (Ac-H4) and total histone H3 (H3) as loading control.

We first investigated the global effect of deacetylase inhibitors on histone H3 and H4 acetylation. Ba/F3 cells were treated for 0, 15, 30 and 60 min with the deacetylase inhibitors that were either effective (200 nM TSA, 3 mM VPA, 500 nM apicidin) or ineffective (1 μM MGCD0103, 5 μM MS-275) in inhibiting STAT5 target gene expression. Histone H3 and H4 acetylation was evaluated by western blot, in parallel to total histone H3 as a control (Figure 5). Interestingly, treatment with TSA, VPA and apicidin, but not with MGCD0103 or MS-275, resulted in a strong increase in histone H3 and H4 acetylation in Ba/F3 cells, in a time-dependent manner. This effect was rapid, detectable already after 15 min treatment. The observation that this rapid increase in histone acetylation correlated with the observed inhibition of STAT5 transactivation properties raises the possibility that these two events are related. It should be noted that the absence of effect of MGCD0103 and MS-275 treatment on global histone H3 and H4 acetylation was likely due to the short duration of treatment applied (up to 90 min). Indeed, increased histone acetylation induced by comparable concentrations of MGCD0103 and MS-275 was demonstrated following 24 h of treatment in various cell lines (71,72). Accordingly, prolonged treatment (10 h) with MS-275 was associated with both increased histone acetylation and reduced *Cis* gene induction (Supplementary Figure S5).

### TSA treatment results in alterations in histone occupancy and acetylation at various gene loci and differentially affects RNA polymerase II recruitment

**Figure 6.**
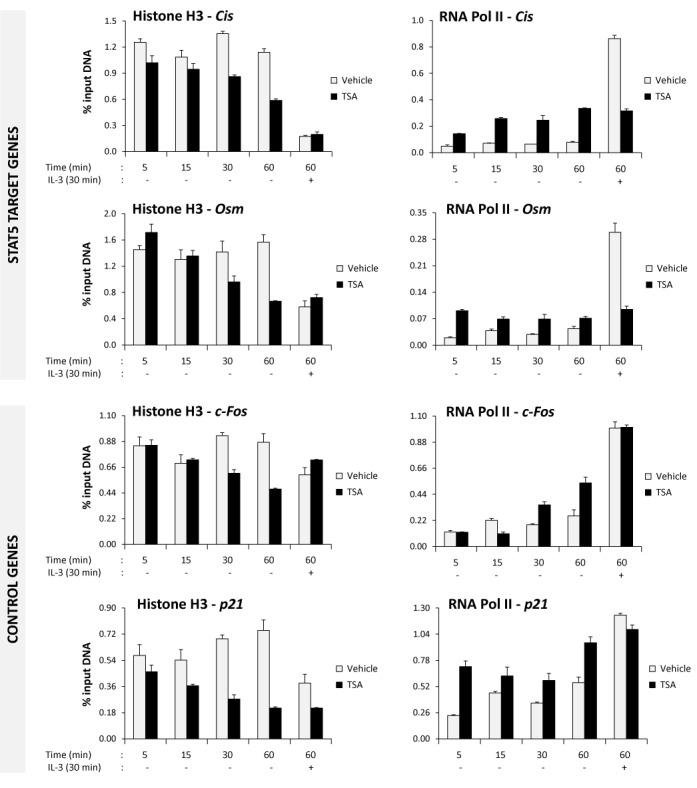
Histone occupancy is reduced at promoters of both STAT5-dependent and -independent genes upon TSA treatment. Ba/F3 cells were withdrawn from IL-3 for 10 h and treated with 200 nM TSA or 0.02% DMSO (vehicle) for 5, 15, 30 and 60 min. As a control for IL-3 stimulation, Ba/F3 cells were stimulated with IL-3 for 30 min following a 30 min TSA pre-treatment (hence treated 60 min with TSA). Cells were harvested and processed for ChIP, as described in Materials and Methods section. ChIP was performed using antibodies directed against total histone H3 or RNA polymerase II (RNA Pol II). Co-precipitated genomic DNA was analysed by quantitative PCR using primers specific for proximal promoter regions (histone H3 ChIP) or transcription start sites (RNA Pol II ChIP) of STAT5 target (*Cis, Osm*) and control (*c-Fos, p21*) genes. *Cis* and *Osm* promoter amplicons overlap their respective STAT5-responsive elements (Supplementary Table S1).

The observed drastic increase in global histone acetylation might have consequences on chromatin organization. Thus, ChIP experiments were performed to analyse histone H3 occupancy, histone H3 and H4 acetylation and RNA polymerase II recruitment at the STAT5 target genes *Cis* and *Osm* and at the control genes *c-Fos* and *p21* (40) following treatment with TSA. Both *c-Fos* and *p21* are IL-3 inducible STAT5-independent genes whose expression is upregulated upon treatment with deacetylase inhibitors via a local increase in histone acetylation (40,41,73). We first investigated the possible occurrence of chromatin alterations upon treatment of unstimulated cells with TSA, that is prior to STAT5 activation. Ba/F3 cells were withdrawn from IL-3 as usual and treated with 200 nM TSA or vehicle for 5, 15, 30 and 60 min. After 30 min TSA pre-treatment, some cells were also stimulated with IL-3 for 30 min. Histone H3 occupancy around the STAT5 binding sites of *Cis* and *Osm* (within their proximal promoters) and the proximal promoters of *c-Fos* and *p21*, as well as RNA polymerase II association with the transcription start site (TSS) of the respective genes, were analysed by ChIP (Figure 6). Interestingly, the amount of histone H3 detected at the proximal promoters of all four genes was reduced upon TSA treatment in a time-dependent manner. This reduction in histone H3 occupancy might reflect a global nucleosome instability following increased histone acetylation, in agreement with previous reports (74–79). Of note, a decrease in histone H3 was also detected at all four proximal promoters upon IL-3 stimulation, suggesting that chromatin remodeling events are also taking place at these IL-3-induced genes, regardless of STAT5 implication. In accordance with our previous reports (40,41), IL-3-induced recruitment of RNA polymerase II at the STAT5 target genes *Cis* and *Osm* was abrogated in the presence of TSA, while it remained unaffected at the control genes *c-Fos* and *p21* (Figure 6). In addition, an increase in RNA polymerase II association was detected at all four genes in unstimulated cells following TSA treatment. This increase is likely the consequence of an enhanced chromatin accessibility following increased histone acetylation and nucleosome loss (74–79). However, RNA polymerase II occupancy in TSA-treated unstimulated cells remained reproducibly below the level reached in vehicle-treated IL-3-stimulated cells in the cases of *Cis* and *Osm*, by contrast to the control genes *c-Fos* and *p21* (Figures 6 and 8), suggesting a distinct mode of recruitment of RNA polymerase II on both sets of genes. Together, these data demonstrate that, concomitantly to a global increase in histone acetylation, TSA treatment results in similar alterations in chromatin structure at multiple genes—as evidenced by a local loss in histone H3 occupancy—and differentially affects RNA polymerase II recruitment at STAT5 target and control genes. Our data therefore raise the possibility that recruitment of RNA polymerase II at STAT5 target genes might require a chromatin-associated factor(s) which might be lost following TSA-induced chromatin reorganization. Noteworthy, these TSA-induced chromatin changes occur already in unstimulated cells, in the absence—hence independently—of nuclear STAT5.

**Figure 7.**
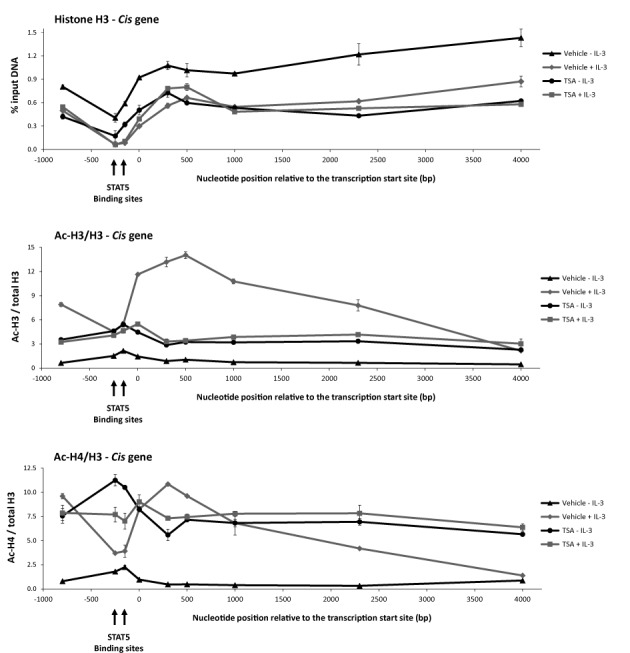
Histone occupancy is reduced and histone acetylation is altered all along the *Cis* gene upon TSA treatment. Rested Ba/F3 cells were pre-treated 30 min with 200 nM TSA or 0.02% DMSO (vehicle) and further stimulated 30 min with IL-3 before being processed for ChIP using antibodies directed against total histone H3, acetylated histone H3 (Ac-H3) and acetylated histone H4 (Ac-H4). Co-precipitated genomic DNA was analysed by quantitative PCR using primers specific for the -800 to +4000 *Cis* gene locus (Supplementary Table S1). Positions of amplicons investigated and of the four STAT5 binding sites arranged in two clusters are indicated along the *x* axis. Non-normalized Ac-H3 and Ac-H4 ChIP data are shown in Supplementary Figure S6.

**Figure 8.**
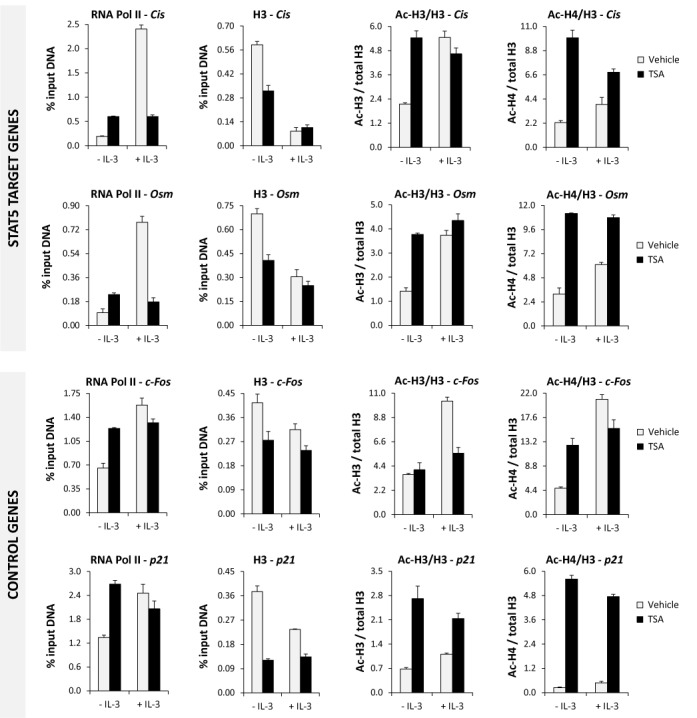
Alterations in histone acetylation by TSA at different gene loci differentially affect gene activation. Rested Ba/F3 cells were pre-treated 30 min with 200 nM TSA or 0.02% DMSO (vehicle) and further stimulated 30 min with IL-3 before being processed for ChIP using antibodies directed against RNA polymerase II (RNA Pol II), histone H3, acetylated histone H3 (Ac-H3) and acetylated histone H4 (Ac-H4), as before. Co-precipitated genomic DNA was analysed by quantitative PCR at STAT5 target (*Cis, Osm*) and control (*c-Fos, p21*) genes, using the same primers as in Figure 6. Non-normalized Ac-H3 and Ac-H4 ChIP data are shown in Supplementary Figure S7.

To better characterize the extent of TSA-induced chromatin alterations, we next investigated histone H3 occupancy and histone H3 and H4 acetylation (Ac-H3 and Ac-H4 respectively) along the *Cis* gene locus. Ba/F3 cells were pre-treated 30 min with TSA and further stimulated with IL-3 for 30 min. Following histone H3, Ac-H3 and Ac-H4 ChIPs, co-precipitated genomic DNA was analysed by quantitative PCR using primers covering the -800 to +4000 region (relative to the transcription start site) of the *Cis* gene (Figure 7 and Supplementary Figure S6). Histone H3 occupancy was lowest around the STAT5 binding sites and decreased equally all along the gene locus upon activation by IL-3 (Figure 7). Following TSA treatment of unstimulated cells, histone H3 level decreased uniformly along the gene and remained unchanged upon further stimulation with IL-3. The fact that the TSA-dependent loss of histone H3 is not further increased upon IL-3 stimulation correlates with the observed inhibition of IL-3-dependent *Cis* expression in TSA-treated cells. To better evaluate changes in histone acetylation following TSA treatment, Ac-H3 and Ac-H4 signals (Supplementary Figure S6) were normalized to histone H3 levels (Figure 7). In unstimulated cells, histone H3 and H4 acetylation was low all along the *Cis* gene locus. Upon IL-3 stimulation, H3 and H4 acetylation increased in the promoter and ORF—in particular within the 5′ region—but remained low at the STAT5 binding sites, in agreement with our previous data (41). Histone H3 and especially histone H4 acetylation was evenly increased along the *Cis* gene in unstimulated TSA-treated cells and did not markedly change following IL-3 stimulation. Ac-H3 and Ac-H4 pattern in TSA-treated cells (repressed gene) were distinct from those observed in untreated IL-3-stimulated cells (activated gene). Our data therefore revealed that (i) IL-3-induced *Cis* gene expression correlates with an increase in histone H3 and H4 acetylation and a nucleosome loss all along the gene locus, and that (ii) TSA treatment results in a nucleosome loss as well, but in altered histone H3 and H4 acetylation patterns.

Similar changes in histone acetylation and H3 loss were found not only at the two STAT5 target genes *Cis* and *Osm* but also at the two control genes *c-Fos* and *p21* investigated (Figure 8 and Supplementary Figure S7). Our data therefore suggest that deacetylase inhibitors such as TSA induce a rapid and global increase in histone acetylation, which results in alterations in nucleosome occupancy and histone acetylation at numerous loci, but has different consequences for the transcription of specific genes. These differences point to chromatin-associated factors, in particular those implicated in the recruitment of the transcriptional machinery, whose function might be affected due to changes in histone acetylation following TSA treatment.

### STAT5-mediated transcription is inhibited by the small-molecule inhibitor of BET family members (+)-JQ1

**Figure 9.**
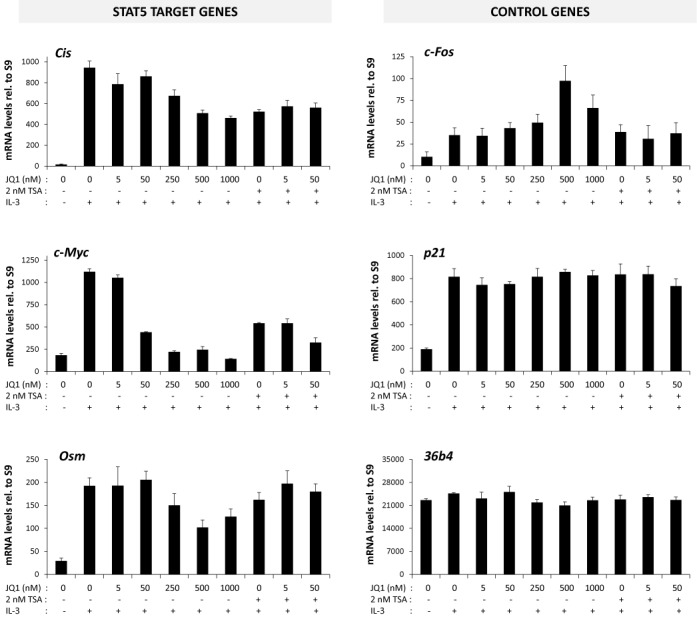
The inhibitor of BET proteins JQ1 inhibits STAT5 activity. Rested Ba/F3 cells were pre-treated 30 min with the indicated concentrations of JQ1, TSA or vehicle (0.02% DMSO in all conditions) and further stimulated 60 min with IL-3. Expression of STAT5 target (*Cis, c-Myc, Osm*) and control (*c-Fos, p21, 36b4*) genes was evaluated by quantitative RT-PCR, as before.

One candidate for a chromatin-associated factor interacting with both acetylated histones and the transcriptional machinery (TBP, RNA polymerase II) is Brd2, which was recently implicated in the regulation of STAT5 activity in leukemia cell lines (42). Brd2 belongs to the BET proteins. The mammalian BET family comprises beside Brd2 the Brd3, Brd4 and BrdT proteins. BET family proteins are transcriptional regulators containing a double bromodomain at their amino-terminus and an extra-terminal protein–protein interaction domain in their carboxy-terminal region (80). They bind to acetylated histones and regulate transcription through interactions with transcription factors, multiple chromatin-modifying enzymes and components of the transcriptional machinery such as TBP and RNA polymerase II (80–86). In support to a role of Brd2 in STAT5-dependent transcriptional regulation in Ba/F3 cells, expression of the STAT5 target genes *Cis, Osm* and *c-Myc* in response to IL-3 was inhibited by the BET-specific inhibitor (+)-JQ1 (thereafter referred to as JQ1) in a dose-dependent manner (Figure 9). In contrast, expression of the control genes *c-Fos, p21* and *36b4* was either increased or remained unaffected (Figure 9). However, no cooperative effect between JQ1 and TSA was found in the presence of sub-optimal concentrations of both compounds (Figure 9), suggesting that both compounds exert partially overlapping effects.

### The BET protein Brd2 is bound to the actively transcribed *Cis* gene and is delocalized upon TSA treatment, presumably to hyperacetylated chromatin

**Figure 10.**
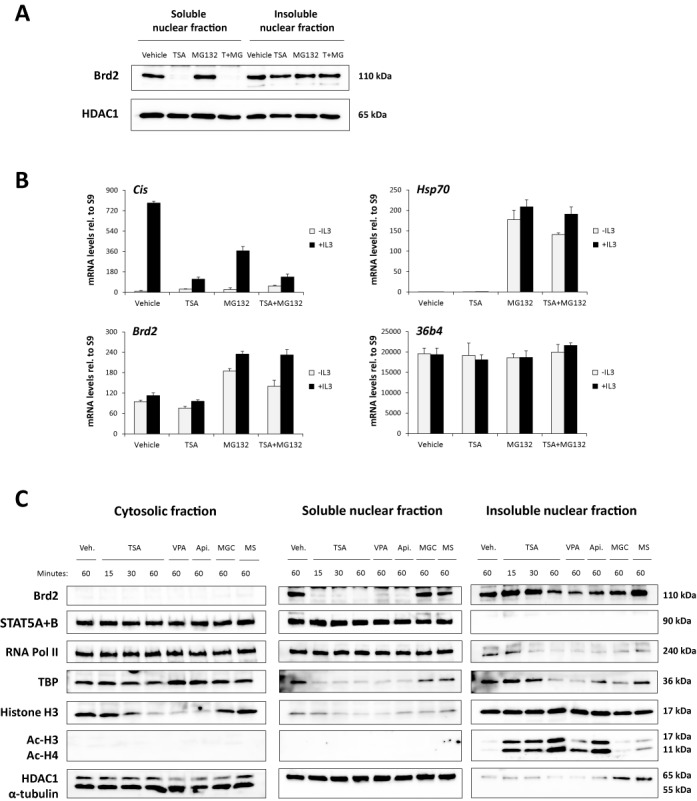
The nuclear BET protein Brd2 is rapidly relocalized to the chromatin fraction together with acetylated histones upon treatment with deacetylase inhibitors that inhibit STAT5 activity. (**A**) Ba/F3 cells were treated with 200 nM TSA, 10 μM MG132, both (T+MG) or vehicle (0.12% DMSO final in all conditions). Cells were treated for a total duration of ∼2 h, with a 45 min MG132 pre-treatment followed by a 90 min TSA treatment. Soluble and insoluble nuclear fractions were analysed by western blot using antibodies against Brd2 and the nuclear marker HDAC1 as loading control. Similar results were obtained upon 3 h pre-treatment with 10 μM MG132 followed by 90 min incubation with 200 nM TSA (4.5 h treatment in total; not shown). (**B**) Ba/F3 cells were rested for 6 h and stimulated with IL-3 for 60 min. Prior to IL-3 stimulation, cells were pre-treated 3.5 h with 10 μM MG132, 30 min with 200 nM TSA, both (3h MG132 followed by 30 min TSA) or vehicle (0.12% DMSO final in all conditions). Hence cells were incubated 4.5 h in total with 10 μM MG132 and 90 min with 200 nM TSA. Expression of the STAT5 target gene *Cis*, of the MG132-regulated gene *hsp70* and of *Brd2* and *36b4* genes as controls was monitored by quantitative RT-PCR. (**C**) Ba/F3 cells were treated for the indicated times with 200 nM TSA, 3 mM valproic acid (VPA), 500 nM apicidin (Api.), 1 μM MGCD0103 (MGC), 5 μM MS-275 (MS), or vehicle (Veh.; 0.02% DMSO final in all conditions). Cytosolic as well as soluble and insoluble nuclear fractions were analysed by western blot using the indicated antibodies. As before, α-tubulin and HDAC1 served as cytosolic and nuclear markers, respectively, to control cell fractionation.

Since the above results suggested a possible implication of Brd2 in STAT5-mediated transcription in Ba/F3 cells, we investigated whether and how TSA could interfere with Brd2 function. We first assessed the subcellular localization of Brd2 in TSA-treated and untreated Ba/F3 cells by cell fractionation and western blot analysis (Figure 10). Brd2 was exclusively detected in nuclear extracts of Ba/F3 cells (Figure 10C), within both the soluble and insoluble (i.e. mainly chromatin-associated) nuclear fractions (Figure 10A and C). Remarkably, upon TSA treatment, Brd2 protein was depleted from the soluble nuclear fraction while it was still detectable in the insoluble nuclear fraction (Figure 10A and C). The depletion of Brd2 from the soluble nuclear fraction was not due to proteasome-dependent protein degradation since it was not prevented by pre-treatment with the proteasome inhibitor MG132 (Figure 10A). MG132 activity was verified by measuring the mRNA levels of *hsp70* (Figure 10B), an acknowledged MG132-induced gene (87). The drop in the level of soluble nuclear Brd2 protein in TSA-treated cells was also not the consequence of a reduced *Brd2* gene expression, since Brd2 mRNA levels were not affected by TSA (Figure 10B). These observations suggest that delocalization of nuclear Brd2 from the soluble to the insoluble chromatin fraction following TSA treatment could limit its availability to support STAT5-mediated transcription.

To address that possibility, Brd2 nuclear distribution was evaluated in Ba/F3 cells treated for 15, 30 and 60 min with 200 nM TSA, as well as for 60 min with the deacetylase inhibitors impairing (3 mM VPA, 500 nM apicidin) or do not impairing (1 μM MGCD0103, 5 μM MS-275) STAT5 target gene expression (Figure 10C). Brd2 loss from the soluble nuclear fraction was already evident after 15 min of TSA treatment, and was similarly observed upon treatment with VPA and apicidin, but not with MGCD0103 or MS-275. Strikingly, Brd2 depletion from the soluble nuclear fraction correlated with the rapid increase in histone H3 and H4 acetylation, which was exclusively detected within the insoluble chromatin fraction (Figure 10C). Of note, the soluble cytosolic pool of free histone H3 (88,89) decreased as the chromatin-bound pool of acetylated histones increased, suggesting that histones are rapidly incorporated into the chromatin upon acetylation induced by TSA, VPA and apicidin. Given that histone H3 occupancy decreased simultaneously at the various gene loci investigated, our data suggest a major redistribution of histone H3 within the chromatin upon deacetylase inhibitor treatment. Brd2 depletion also correlated with a specific decrease of TBP in the soluble nuclear fraction, suggesting that—concomitantly to Brd2—TBP is also redistributed to hyperacetylated chromatin. This is in agreement with the reported interaction between Brd2, acetylated histones and TBP (82,83,85). STAT5 and RNA polymerase II showed no apparent depletion from the soluble nuclear fraction upon deacetylase inhibitor treatment (Figure 10C). Overall, these experiments indicate that deacetylase inhibitors such as TSA, VPA and apicidin—but not MGCD0103 or MS-275—induce a rapid increase in chromatin acetylation that might result in a massive delocalization of Brd2 and of Brd2-associated TBP, likely due to Brd2 preferential binding to hyperacetylated chromatin (82,83,85). Together with the finding that the BET-specific inhibitor JQ1 inhibits STAT5 target gene expression in IL-3-stimulated Ba/F3 cells (Figure 9), our data suggest that Brd2 is required for efficient STAT5-mediated transcription and that its rapid redistribution following treatment with TSA, VPA or apicidin is responsible for the inhibition of STAT5 transcriptional activity.

**Figure 11.**
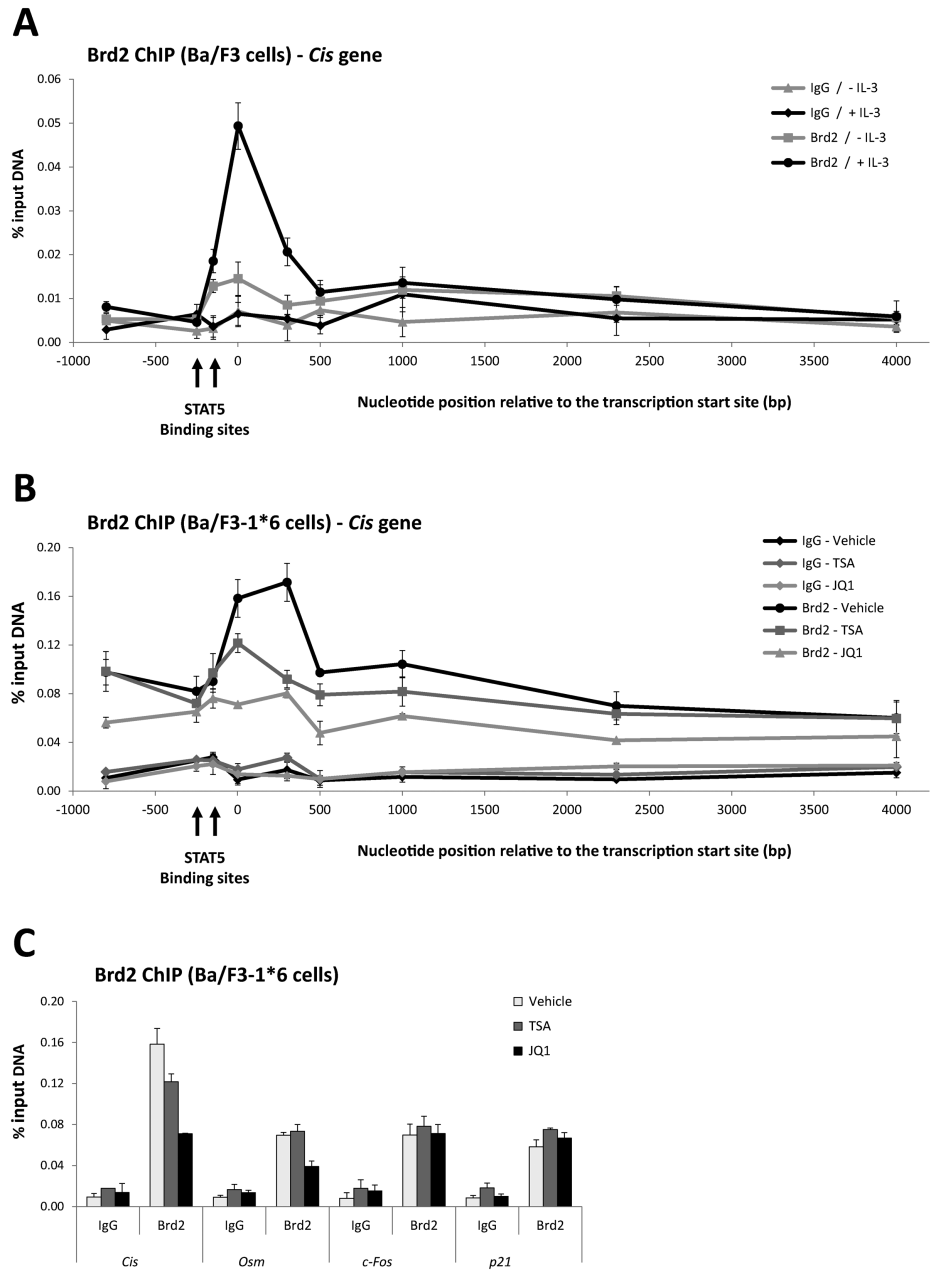
Brd2 protein is present at the transcriptionally active *Cis* locus and is lost following both TSA and JQ1 treatment. (**A**) Rested Ba/F3 cells were either kept unstimulated (- IL-3; STAT5 activity off) or stimulated with IL-3 for 30 min (+ IL-3; STAT5 activity turned on). Brd2 ChIP was performed on sonified whole cell extracts from formaldehyde-cross-linked cells using 3 μg of either Brd2-specific antibodies or rabbit IgG (background control). Co-precipitated genomic DNA was analysed by quantitative PCR using the same *Cis* primers as in Figure 7. (**B, C**) Ba/F3–1*6 cells were treated for 60 min with 200 nM TSA, 1 μM JQ1 or vehicle (0.02% DMSO). Brd2 ChIP was performed on sonified nuclear extracts from formaldehyde-cross-linked cells as described in Materials and Methods section using 3 μg of either Brd2-specific antibodies or rabbit IgG. Co-precipitated genomic DNA was analysed by quantitative PCR using the same primers as in panel A (**B**) or using primers specific for the transcription start site of STAT5 target (*Cis, Osm*) and control (*c-Fos, p21*) genes (Supplementary Table S1) (**C**).

This model predicts that Brd2 is associated with actively transcribed STAT5 target genes and that this association is lost upon TSA (or JQ1) treatment. To validate this model, Brd2 ChIP assays were conducted in unstimulated versus IL-3-stimulated Ba/F3 cells as well as in Ba/F3–1*6 cells treated with 200 nM TSA, 1 μM JQ1 or DMSO (vehicle). Brd2 association with the *Cis* gene locus was investigated using primers covering the -800/+4000 region, as before (Figure 11A and B). Upon IL-3 stimulation of Ba/F3 cells, Brd2 protein was recruited to the transcription start site of the *Cis* gene (Figure 11A), supporting the idea that binding of Brd2 is STAT5-dependent. Similarly, in untreated Ba/F3–1*6 cells, Brd2 protein was bound at the transcription start site and 5′ region of the open reading frame of the transcriptionally active *Cis* gene. As predicted, Brd2 association with the *Cis* gene was strongly reduced upon treatment with either TSA or JQ1 (Figure 11B and C). By contrast, Brd2 protein level detected at the transcription start site of the control genes *c-Fos* and *p21* was low and remained unaffected by TSA or JQ1 treatment (Figure 11C). Noticeably, the level of Brd2 protein detected at the transcription start site of the STAT5 target gene *Osm* was also low and was reduced in JQ1—but not in TSA-treated cells.

In summary, we showed that the deacetylase inhibitors TSA, VPA and apicidin—but not MGCD0103 or MS-275—inhibit STAT5-mediated transcription in correlation with alterations of histone occupancy and acetylation, but likely independently of STAT5 acetylation. The BET protein inhibitor JQ1 also inhibits STAT5-mediated transcription, suggesting a connection between BET proteins and STAT5-dependent activation of transcription. Accordingly, the BET protein Brd2 was bound at the transcriptionally active *Cis* gene and was lost upon both JQ1 and TSA treatment. Brd2 loss from the *Cis* gene following TSA treatment is possibly the consequence of a vast depletion of Brd2 from the nucleosol due to its preferential interaction with hyperacetylated chromatin, which is induced in response to TSA treatment. Nucleosolic levels of TBP, a Brd2 interacting partner, were also reduced upon Brd2 depletion, suggesting that TBP is co-delocalized with Brd2. It also raises the possibility that one of Brd2 functions is to assist recruitment and/or stabilization of the transcriptional machinery at STAT5 target genes. Altogether, our data suggest that the loss of Brd2—and possibly of other BET proteins—associated with TSA treatment is responsible for the impaired assembly of the transcriptional machinery and the resulting transcriptional inhibition at STAT5 target genes.

## DISCUSSION

We showed before that the deacetylase inhibitors TSA, SAHA and NaB inhibit STAT5 transcriptional activity at a step following STAT5 activation and binding to DNA by preventing recruitment of the transcriptional machinery (40). The effect of deacetylase inhibitor treatment on local histone acetylation at the STAT5 target genes was marginal (40,41) and convinced us to consider the possibility that inhibition of STAT5 activity by deacetylase inhibitors targets STAT5 protein rather than chromatin.

We now show that the effect of deacetylase inhibitors on STAT5 activity is independent of STAT5 acetylation. Mutation of lysine residues previously shown to be acetylated within STAT5 not only did not affect expression of endogenous STAT5 target genes in Ba/F3 cells but also did not suppress the sensitivity to TSA. STAT5 acetylation was previously shown to take place in the cytosol and play a role in its dimerization (10). Our results using constitutively active STAT5A-1*6 further demonstrate that STAT5 acetylation is likely required for its initial activation (phosphorylation, dimerization, nuclear translocation)—a step circumvented using constitutively active STAT5A-1*6—but not for its transcriptional activity. This might explain why transactivation of a luciferase reporter by a STAT5A K696R mutant was partially impaired in GH-stimulated mouse embryonic fibroblasts (MEFs) (8), likely as a result of a defective STAT5 activation rather than a transcriptional defect.

Instead, we found that the deacetylase inhibitors TSA, VPA and apicidin, but not MGCD0103 and MS-275, induce a rapid increase in global histone H3 and H4 acetylation, which correlates with their ability to suppress STAT5-mediated transcription. Our data suggest that the absence of effect of MGCD0103 and MS-275 on global histone H3 and H4 acetylation is the consequence of a short treatment duration rather than of compound inactivity. We also found that the BET protein inhibitor (+)-JQ1 prevents induction of STAT5 target genes in Ba/F3 cells and that the BET protein Brd2 is associated with the transcriptionally active *Cis* gene in a STAT5-dependent manner, supporting a role of Brd2 in STAT5-mediated transcription. This is in agreement with the recent demonstration that BET proteins, in particular Brd2, regulate STAT5 activity in human leukemia cells (42). Unexpectedly, we did not observe a synergy between JQ1 and TSA in inhibiting expression of STAT5 target genes in Ba/F3 cells, which would be anticipated for inhibitors targeting the same factor. On the other hand, this observation is in agreement with several recent reports showing that JQ1 and the pan-deacetylase inhibitor panobinostat (or tyrosine kinase inhibitors) synergistically induce apoptosis of leukemia cells expressing constitutive active STAT5 but not of normal hematopoietic progenitor cells (42,48,49). A lack of synergy between JQ1 and TSA might indicate that the effect of JQ1 only partially overlaps that of TSA. For instance, although both JQ1 and TSA alter Brd2 function, JQ1 does not affect histone acetylation, as opposed to TSA.

The mechanism of STAT5-dependent recruitment of Brd2 to the *Cis* gene is still unknown. A direct interaction between STAT5 and Brd2 could not be demonstrated in co-immunoprecipitation experiments (data not shown), suggesting that this event takes place at the chromatin level rather than in solution. The proposition that STAT5 and Brd2 are unlikely to form a stable complex in solution is also supported by our observations that TSA treatment has no effect on (i) the pool of nucleosolic STAT5 detected in western blot upon Brd2 depletion from the nucleosol, (ii) STAT5 binding to DNA in chromatin immunoprecipitations (this study and (40,41)) and (iii) the elution profile of STAT5-containing complexes in gel filtration.

Importantly, we found that Brd2 dissociates from the *Cis* gene upon TSA treatment, in correlation with Brd2 depletion from the nucleosol as chromatin becomes hyperacetylated. Since Brd2 is known to associate preferentially with hyperacetylated histones (85), our data suggest that Brd2 is rapidly delocalized to acetylated chromatin upon treatment with deacetylase inhibitors. No increase in Brd2 protein level was detected in the insoluble chromatin fraction as nucleosolic Brd2 decreased, which would have been expected upon Brd2 redistribution. This might be inherent to the method of protein extraction that we used. We tried but failed to achieve total protein extraction from the insoluble nuclear fraction (see Materials and Methods section), and thus it is possible that part of the chromatin-bound proteins were not loaded on SDS-PAGE. Nevertheless, since we demonstrated that Brd2 depletion was not due to protein degradation and since the level of Brd2-associated protein TBP (82,83) decreased concomitantly, our data strongly suggest that Brd2 is delocalized, rather than degraded, as a consequence of its preferential association with hyperacetylated histones. Such a mechanism might explain why we failed to identify the HDAC(s) implicated in STAT5 target gene expression in our siRNA transfection experiments, since global histone acetylation was only marginally affected in these experiments. Along these lines, the huge increase in histone acetylation observed upon treatment with TSA, VPA or apicidin suggests that not one but several HDACs are simultaneously targeted. The use of specific deacetylase inhibitors might help addressing that point. On the other hand, the observation that the chemical inhibition and physical elimination of HDACs do not have the same effect on STAT5 target gene expression might reflect a distinct mode of action of HDACs, possibly independent of their deacetylase activity, for instance involving HDAC inhibitor-induced protein complex disruption, in agreement with previous reports (90–94).

Noticeably, although *Osm* gene expression was reduced upon JQ1 treatment, the level of Brd2 protein detected at the transcription start site was lower than that detected at the *Cis* gene and was not reduced upon TSA treatment, indicating that Brd2 is not involved in the regulation of *Osm*. This suggests that other members of the BET family, such as Brd3 or Brd4 (80), might also be involved in the regulation of STAT5 target gene expression.

Brd2 protein interacts with a number of transcription factors, chromatin-modifying factors and components of the transcriptional machinery including TBP and RNA polymerase II (82,83,86). One can thus anticipate that the abrupt redistribution of Brd2 interferes with the proper recruitment of transcription regulatory factors to chromatin. Accordingly, nucleosolic TBP was reduced together with Brd2 depletion upon treatment with TSA, VPA and apicidin. We propose that TSA-dependent depletion of soluble Brd2 interferes with the proper recruitment and/or stabilization of the transcriptional machinery. This is also supported by our observation that TSA treatment of either IL-3-stimulated Ba/F3 cells or Ba/F3–1*6 cells results in a rapid reduction of Cis mRNA levels simultaneously to the dissociation of TBP and RNA polymerase II from the transcription start site ((40) and Figure 1 respectively). Beside their interaction with factors regulating transcription, BET proteins interact with chromatin remodeling complexes and regulate the maintenance of higher-order chromatin structure (81,83,86). Therefore, it is possible that Brd2 delocalization also alters chromatin organization, in agreement with the nucleosome loss monitored at all the investigated genes upon TSA treatment.

In summary, we showed that BET proteins including Brd2 play an important role in the regulation of STAT5 target gene expression, likely by assisting the recruitment and stabilization of the transcriptional machinery. We showed, to our knowledge for the first time, that the function of BET proteins is altered by deacetylase inhibitors, very likely by provoking a mislocalization of Brd2 and possibly of other BET family members to hyperacetylated chromatin (Supplementary Figure S8). Our data thus reveal novel and essential aspects of the mechanism of transcriptional regulation by STAT5 and of its inhibition by deacetylase inhibitors, and underscore BET proteins as potential therapeutic targets in the treatment of STAT5-associated cancers.

## SUPPLEMENTARY DATA

Supplementary Data are available at NAR Online.

SUPPLEMENTARY DATA
