# Supplementary material for: Deacetylase inhibitors repress STAT5-mediated transcription by interfering with bromodomain and extra-terminal (BET) protein function
Source: Nucleic Acids Res. 2015 Mar 13;43(7):3524–45. doi: 10.1093/nar/gkv188 (PMC4402521; doi:10.1093/nar/gkv188)
Supplement: SUPPLEMENTARY DATA [file supp_43_7_3524__index.html]

Deacetylase inhibitors repress STAT5-mediated transcription by interfering with bromodomain and extra-terminal (BET) protein function — Deacetylase inhibitors repress STAT5-mediated transcription by interfering with bromodomain and extra-terminal (BET) protein function — SUPPLEMENTARY DATA 

# Deacetylase inhibitors repress STAT5-mediated transcription by interfering with bromodomain and extra-terminal (BET) protein function

## SUPPLEMENTARY DATA

**Files in this Data Supplement:**

- SUPPLEMENTARY DATA
