# Supplementary material for: Deacetylase inhibitors repress STAT5-mediated transcription by interfering with bromodomain and extra-terminal (BET) protein function
Source: Nucleic Acids Res. 2015 Mar 13;43(7):3524–45. doi: 10.1093/nar/gkv188 (PMC4402521; doi:10.1093/nar/gkv188)
Supplement: SUPPLEMENTARY DATA [file supp_gkv188_nar-03710-x-2014-File013.pdf]

SUPPLEMENTARY DATA

SUPPLEMENTARY FIGURES LEGENDS

Table S1. DNA and RNA oligonucleotides used in this study.

Table S2. IC_{50} reported in the literature for *in vitro* inhibition of recombinant human HDACs by pan- and class I-selective deacetylase inhibitors. The concentration of deacetylase inhibitors necessary for 50% and higher inhibition of STAT5 activity (from Figure 4 and S2) is also indicated.

Figure S1. STAT5-containing complexes show no apparent reorganisation upon trichostatin A (TSA) treatment. Soluble nuclear lysates from Ba/F3-1*6 cells treated 60 min with 200 nM TSA or 0.02% DMSO (vehicle) were subjected to gel filtration chromatography as described in Material and Methods, and eluted fractions were analysed by Western blot using antibodies directed against FLAG (STAT5A-1*6), STAT5A+B (total STAT5) and RNA polymerase II (RNA Pol II). Input lysate (i) and fraction number (no.) are indicated. The protein markers thyroglobulin (669 kDa) and BSA (66 kDa) co-eluted with fractions 13 (top arrow) and 24 (not shown) respectively. The STAT5 protein dimer peak eluted in fraction 20 and no STAT5 signal was detected in fractions 10-12 (not shown). Therefore only fractions 2-9 and 13-20 are shown.

Figure S2. STAT5A activity in Ba/F3 cells is differentially affected by class-selective deacetylase inhibitors. Ba/F3 cells were pre-treated with the indicated concentrations of the pan-inhibitor trichostatin A (TSA) and of the class I-selective deacetylase inhibitors valproic acid (VPA), apicidin, MGCD0103 and MS-275 for 15 min (TSA) or 30 min (all others), and further stimulated with IL-3 for 60 min, as described in Material and Methods. The final concentration of DMSO was adjusted in each experiment as follows: 0.02% for 0-200 nM trichostatin A (TSA), 0-1000 nM apicidin, 0-2500 nM MGCD0103 and for 0-5 µM MS-275; 0.08% for 20 µM MS-275; 0% for 0-30 mM valproic acid (VPA) as vehicle is H_2O. Expression of STAT5 target (*Cis*, *c-Myc*, *Osm*) and control (*c-Fos*, *36b4*) genes was evaluated by quantitative RT-PCR as detailed in Material and Methods.

Figure S3. Expression of lysine deacetylases HDAC1-11 in Ba/F3 cells. Expression of HDAC1 to HDAC11 was monitored by quantitative RT-PCR in Ba/F3 cells both unstimulated and stimulated for 2 h with IL-3. CT values for HDAC9 and HDAC11 were at the limit of detection (not shown).

Figure S4. siRNA-mediated knock-down of HDAC1-11 does not affect STAT5 transcriptional activity. (**A-D**) Ba/F3 cells were electroporated in the presence of 7 µM scramble siRNA (ScI) or siRNA targeting the indicated mouse HDACs (HD), using Bio-Rad Gene Pulser II with RF Module as previously described (1). IL-3 stimulation of transfected cells was performed as described (1). (**E-G**) Ba/F3-1*6 cells were electroporated in the presence of 0.5 to 1 µM of the indicated siRNAs (ScI or HDAC (HD)-specific), using Bio-Rad Gene Pulser Xcell with CE Module as described in Material and Methods. Expression of the STAT5 target genes *Cis* (A-C, E, G) and *c-Myc* (G), of the respective *HDACs* (A-C, E, G) and of the control gene *36b4* (G) was analysed by quantitative RT-PCR as before. HDAC2 mRNA level was measured as an additional control in panels C and E (right section). Immunoblotting of whole-cell Brij lysates was performed to verify the HDAC knock-down efficiency at the protein

level, whenever commercial antibodies functioning in Western blot were available (HDAC1, HDAC2 and HDAC3). In panel F, Histone H3 and H4 acetylation (Ac-H3 and Ac-H4 respectively) and total histone H3 were also monitored. α-tubulin served as a loading control, except in panel D where total STAT5 was used. (*) in panel D designates a non-specific signal. The efficiency of HDAC9 and HDAC11 siRNAs could not be established as the corresponding mRNA levels were at the limit of detection (not shown). Of note, HDAC1 knock-down performed in the triple-transfection shown in panel F was ineffective, possibly due to the lower amount of the individual siRNAs used.

Figure S5. Prolonged treatment with MS-275 also correlates with increased histone acetylation and inhibition of STAT5 activity. (**A**) Ba/F3 cells were treated for 10 h with 5 µM MS-275 or 0.02% DMSO (vehicle). Ba/F3 cells treated for 30 min with 200 nM trichostatin A (TSA) served as a reference for induced histone acetylation. Freeze-thaw protein lysates were analysed by Western blot, as described in Figure 5. (**B**) Ba/F3 cells were withdrawn from IL-3 for 10 h in the presence of 5 µM MS-275 or 0.02% DMSO (vehicle). As a control, Ba/F3 cells were rested in parallel for 10 h and treated for the last 30 min with 200 nM TSA. Rested Ba/F3 cells were stimulated with IL-3 for 30 min and expression of the STAT5 target gene *Cis* and of the control gene *36b4* was analysed by quantitative RT-PCR, as before.

Figure S6. Histone H3 and H4 acetylation along the *Cis* gene upon trichostatin A (TSA) treatment. Rested Ba/F3 cells were pre-treated 30 min with 200 nM TSA or 0.02% DMSO (vehicle) and further stimulated 30 min with IL-3 before being processed for chromatin immunoprecipitation (ChIP) using antibodies directed against acetylated histone H3 (Ac-H3) and acetylated histone H4 (Ac-H4). Co-precipitated genomic DNA was analysed by quantitative PCR using primers specific for the -800 to +4000 *Cis* gene locus (Table S1). Position of amplicons investigated and of the four STAT5 binding sites are indicated along the x axis. The corresponding data normalized to histone H3 occupancy are shown in Figure 7.

Figure S7. Alterations in histone H3 and H4 acetylation by trichostatin A (TSA) at STAT5 target (*Cis*, *Osm*) and control (*c-Fos*, *p21*) genes. Rested Ba/F3 cells were pre-treated 30 min with 200 nM TSA or 0.02% DMSO (vehicle) and further stimulated 30 min with IL-3 before being processed for chromatin immunoprecipitation (ChIP) using antibodies directed against acetylated histone H3 (Ac-H3) and acetylated histone H4 (Ac-H4), as before. Co-precipitated genomic DNA was analysed by quantitative PCR using the same primers as in Figure 6. The corresponding data normalized to histone H3 occupancy are shown in Figure 8.

Figure S8. Model of implication of BET proteins in STAT5-mediated transcription of the *Cis* gene and of its repression by deacetylase inhibitors. Brd2 proteins are normally present in both the soluble and insoluble (i.e. chromatin-associated) nuclear compartments in Ba/F3-derived cells (**A**, **B**). STAT5 activation (i.e. wild-type STAT5 in IL-3-stimulated Ba/F3 cells or constitutively active STAT5 in Ba/F3-1*6 cells) results in the recruitment of Brd2 and of the pre-initiation complex (PIC) to the transcription start site of the *Cis* gene, leading to transcriptional gene activation (**B**). Treatment with deacetylase inhibitors such as trichostatin A (TSA), apicidin or valproic acid results in a rapid chromatin hyperacetylation associated with the depletion of Brd2 from the nucleosol due to its preferential binding to acetylated histones (**C**). Together with our previous demonstration that TSA does not affect STAT5 binding to DNA but prevents the proper recruitment of the transcriptional

machinery (27), our data suggest that the loss of Brd2 from the *Cis* promoter is responsible for the failed recruitment and/or destabilisation of the PIC, and thus for the transcriptional repression of the *Cis* gene that occurs following TSA treatment (**C**). The HDAC(s) involved in the proposed model remain(s) to be identified. Also, we cannot rule out that, beside histone hyperacetylation, a deacetylase activity-independent mechanism is involved in regulating Brd2 function. Finally, our data suggest that, beside Brd2, other chromatin-associated factors might be involved in the regulation of STAT5 activity and its inhibition by deacetylase inhibitors.

REFERENCES TO SUPPLEMENTARY INFORMATION (INCLUDING TABLE S2)

1. Rascle,A. and Lees,E. (2003) Chromatin acetylation and remodeling at the Cis promoter during STAT5-induced transcription. *Nucleic Acids Res.*, **31**, 6882–6890.

2. Arts,J., Angibaud,P., Mariën,A., Floren,W., Janssens,B., King,P., van Dun,J., Janssen,L., Geerts,T., Tuman,R.W., *et al.* (2007) R306465 is a novel potent inhibitor of class I histone deacetylases with broad-spectrum antitumoral activity against solid and haematological malignancies. *Br. J. Cancer*, **97**, 1344–1353.

3. Khan,N., Jeffers,M., Kumar,S., Hackett,C., Boldog,F., Khramtsov,N., Qian,X., Mills,E., Berghs,S.C., Carey,N., *et al.* (2008) Determination of the class and isoform selectivity of small-molecule histone deacetylase inhibitors. *Biochem. J.*, **409**, 581–589.

4. Estiu,G., West,N., Mazitschek,R., Greenberg,E., Bradner,J.E. and Wiest,O. (2010) On the inhibition of histone deacetylase 8. *Bioorg. Med. Chem.*, **18**, 4103–4110.

5. Marek,L., Hamacher,A., Hansen,F.K., Kuna,K., Gohlke,H., Kassack,M.U. and Kurz,T. (2013) Histone deacetylase (HDAC) inhibitors with a novel connecting unit linker region reveal a selectivity profile for HDAC4 and HDAC5 with improved activity against chemoresistant cancer cells. *J. Med. Chem.*, **56**, 427–436.

6. Oehme,I., Deubzer,H.E., Lodrini,M., Milde,T. and Witt,O. (2009) Targeting of HDAC8 and investigational inhibitors in neuroblastoma. *Expert Opin. Investig. Drugs*, **18**, 1605–1617.

7. Vannini,A., Volpari,C., Filocamo,G., Casavola,E.C., Brunetti,M., Renzoni,D., Chakravarty,P., Paolini,C., De Francesco,R., Gallinari,P., *et al.* (2004) Crystal structure of a eukaryotic zinc-dependent histone deacetylase, human HDAC8, complexed with a hydroxamic acid inhibitor. *Proc. Natl. Acad. Sci. U. S. A.*, **101**, 15064–15069.

8. Jeon,H.S., Ahn,M.Y., Park,J.H., Kim,T.H., Chun,P., Kim,W.H., Kim,J., Moon,H.R., Jung,J.H. and Kim,H.S. (2010) Anticancer effects of the MHY218 novel hydroxamic acid-derived histone deacetylase inhibitor in human ovarian cancer cells. *Int. J. Oncol.*, **37**, 419–428.

9. Hu,E., Dul,E., Sung,C.-M., Chen,Z., Kirkpatrick,R., Zhang,G.-F., Johanson,K., Liu,R., Lago,A., Hofmann,G., *et al.* (2003) Identification of novel isoform-selective inhibitors within class I histone deacetylases. *J. Pharmacol. Exp. Ther.*, **307**, 720–728.

10. Hess-Stumpp,H., Bracker,T.U., Henderson,D. and Politz,O. (2007) MS-275, a potent orally available inhibitor of histone deacetylases--the development of an anticancer agent. *Int. J. Biochem. Cell Biol.*, **39**, 1388–1405.

11. Guardiola,A.R. and Yao,T.-P. (2002) Molecular cloning and characterization of a novel histone deacetylase HDAC10. *J. Biol. Chem.*, **277**, 3350–3356.

12. Wagner,F.F., Weïwer,M., Lewis,M.C. and Holson,E.B. (2013) Small molecule inhibitors of zinc-dependent histone deacetylases. *Neurother. J. Am. Soc. Exp. Neurother.*, **10**, 589–604.

13. Arts,J., King,P., Mariën,A., Floren,W., Beliën,A., Janssen,L., Pilatte,I., Roux,B., Decrane,L., Gilissen,R., *et al.* (2009) JNJ-26481585, a novel 'second-generation' oral histone deacetylase inhibitor, shows broad-spectrum preclinical antitumoral activity. *Clin. Cancer Res. Off. J. Am. Assoc. Cancer Res.*, **15**, 6841–6851.

14. Lee,J.-H., Mahendran,A., Yao,Y., Ngo,L., Venta-Perez,G., Choy,M.L., Kim,N., Ham,W.-S., Breslow,R. and Marks,P.A. (2013) Development of a histone deacetylase 6 inhibitor and its biological effects. *Proc. Natl. Acad. Sci. U. S. A.*, **110**, 15704–15709.

15. Sun,Q., Yao,Y., Liu,C., Li,H., Yao,H., Xue,X., Liu,J., Tu,Z. and Jiang,S. (2013) Design, synthesis, and biological evaluation of novel histone deacetylase 1 inhibitors through click chemistry. *Bioorg. Med. Chem. Lett.*, **23**, 3295–3299.

16. Tashima,T., Murata,H. and Kodama,H. (2014) Design and synthesis of novel and highly-active pan-histone deacetylase (pan-HDAC) inhibitors. *Bioorg. Med. Chem.*, **22**, 3720–3731.

17. Balasubramanian,S., Verner,E. and Buggy,J.J. (2009) Isoform-specific histone deacetylase inhibitors: the next step? *Cancer Lett.*, **280**, 211–221.

18. Tang,W., Luo,T., Greenberg,E.F., Bradner,J.E. and Schreiber,S.L. (2011) Discovery of histone deacetylase 8 selective inhibitors. *Bioorg. Med. Chem. Lett.*, **21**, 2601–2605.

19. Tong,J.J., Liu,J., Bertos,N.R. and Yang,X.-J. (2002) Identification of HDAC10, a novel class II human histone deacetylase containing a leucine-rich domain. *Nucleic Acids Res.*, **30**, 1114–1123.

20. Oger,F., Lecorgne,A., Sala,E., Nardese,V., Demay,F., Chevance,S., Desravines,D.C., Aleksandrova,N., Le Guével,R., Lorenzi,S., *et al.* (2010) Biological and biophysical properties of the histone deacetylase inhibitor suberoylanilide hydroxamic acid are affected by the presence of short alkyl groups on the phenyl ring. *J. Med. Chem.*, **53**, 1937–1950.

21. Beckers,T., Burkhardt,C., Wieland,H., Gimmnich,P., Ciossek,T., Maier,T. and Sanders,K. (2007) Distinct pharmacological properties of second generation HDAC inhibitors with the benzamide or hydroxamate head group. *Int. J. Cancer J. Int. Cancer*, **121**, 1138–1148.

22. Jones,P., Altamura,S., Chakravarty,P.K., Cecchetti,O., De Francesco,R., Gallinari,P., Ingenito,R., Meinke,P.T., Petrocchi,A., Rowley,M., *et al.* (2006) A series of novel, potent, and selective histone deacetylase inhibitors. *Bioorg. Med. Chem. Lett.*, **16**, 5948–5952.

23. Gurvich,N., Tsygankova,O.M., Meinkoth,J.L. and Klein,P.S. (2004) Histone deacetylase is a target of valproic acid-mediated cellular differentiation. *Cancer Res.*, **64**, 1079–1086.

24. Göttlicher,M., Minucci,S., Zhu,P., Krämer,O.H., Schimpf,A., Giavara,S., Sleeman,J.P., Lo Coco,F., Nervi,C., Pelicci,P.G., *et al.* (2001) Valproic acid defines a novel class of HDAC inhibitors inducing differentiation of transformed cells. *EMBO J.*, **20**, 6969–6978.

25. Le Tourneau,C. and Siu,L.L. (2008) Promising antitumor activity with MGCD0103, a novel isotype-selective histone deacetylase inhibitor. *Expert Opin. Investig. Drugs*, **17**, 1247–1254.

26. Fournel,M., Bonfils,C., Hou,Y., Yan,P.T., Trachy-Bourget,M.-C., Kalita,A., Liu,J., Lu,A.-H., Zhou,N.Z., Robert,M.-F., *et al.* (2008) MGCD0103, a novel isotype-selective histone deacetylase inhibitor, has broad spectrum antitumor activity in vitro and in vivo. *Mol. Cancer Ther.*, **7**, 759–768.

27. Rascle,A., Johnston,J.A. and Amati,B. (2003) Deacetylase activity is required for recruitment of the basal transcription machinery and transactivation by STAT5. *Mol. Cell. Biol.*, **23**, 4162–4173.

Pinz et al.

Table S1. DNA and RNA Oligonucleotides used

Mouse STAT5A-specific site-directed mutagenesis primers (Source: Metabion)

Mutation		5'-3' sequence (Bold: mutated codon; underlined: screening restriction site, silent mutation)	Template plasmid DNA	Screening restriction enzyme
K84Q	Forward	ATGGGTTTTTGCTG**CAG**ATCAAGCTTGGGCACTATGCCAC	pcDNA3-STAT5A-1*6	HindIII
	Reverse	GTGGCATAGTGCCCAAGCTTGAT**CTG**CAGCAAAAACCCAT		
K84R	Forward	GGGAAGATGGGTTTTTGCTG**AGG**ATCAAGCTTGGGCACTATGC	pcDNA3-STAT5A-1*6	HindIII
	Reverse	GCATAGTGCCCAAGCTTGAT**CCT**CAGCAAAAACCCATCTTCCC		
K359Q	Forward	CCACCGTGCGCCTACTAGTGGGGGGA**CAG**CTGAATG	pcDNA3-STAT5A-1*6	SpeI
	Reverse	CATTCAG**CTG**TCCCCCCACTAGTAGGCGCACGGTGG		
K359R	Forward	CACCGTGCGCCTACTAGTGGGGGGA**AGG**CTGAATGTG	pcDNA3-STAT5A-1*6	SpeI
	Reverse	CACATTCAG**CCT**TCCCCCCACTAGTAGGCGCACGGTG		
K384Q	Forward	GTCCCTGCTC**CAG**AATGAGAACACGCGTAATGAGTGCAGC	pcDNA3-STAT5A-1*6	MluI
	Reverse	GCTGCACTCATTACGCGTGTTCTCATT**CTG**GAGCAGGGAC		
K384R	Forward	GTCCCTGCTC**AGG**AATGAGAACACGCGTAATGAGTGCAGC	pcDNA3-STAT5A-1*6	MluI
	Reverse	GCTGCACTCATTACGCGTGTTCTCATT**CCT**GAGCAGGGAC		
K675Q	Forward	TTATCTACGTGTTCCCCGATCGACCC**CAG**GACGAGGTCTTTGC	pcDNA3-STAT5A-1*6	PvuI
	Reverse	GCAAAGACCTCGTC**CTG**GGGTCGATCGGGGAACACGTAGATAA		
K675R	Forward	CTTATCTACGTGTTCCCCGATCGACCC**CGG**GACGAGGTCTTTGCCA	pcDNA3-STAT5A-1*6	PvuI
	Reverse	TGGCAAAGACCTCGTC**CCG**GGGTCGATCGGGGAACACGTAGATAAG		
K681Q	Forward	AGGACGAGGTCTTTGCC**CAG**TACTACACTCCTGTACTTGC	pcDNA3-STAT5A-1*6	ScaI
	Reverse	GCAAGTACAGGAGTGTAGTA**CTG**GGCAAAGACCTCGTCCT		
K681R	Forward	ACGAGGTCTTTGCC**CGG**TATTACACTCCAGTACTTGCGAAAG	pcDNA3-STAT5A-1*6	ScaI
	Reverse	CTTTCGCAAGTACTGGAGTGTAATA**CCG**GGCAAAGACCTCGT		
K689Q	Forward	CCAAGTATTACACTCCAGTACTTGCG**CAA**GCAGTTGACGGATA	pcDNA3-STAT5A-1*6	ScaI
	Reverse	TATCCGTCAACTGC**TTG**CGCAAGTACTGGAGTGTAATACTTGG		
K689R	Forward	GCCAAGTATTACACTCCAGTACTTGCG**CGA**GCAGTTGACGGATACG	pcDNA3-STAT5A-1*6	ScaI
	Reverse	CGTATCCGTCAACTGC**TCG**CGCAAGTACTGGAGTGTAATACTTGGC		
Y694F	Forward	CCTGTACTTGCGAAAGCAGTCGACGGA**TTC**GTGAAGCCAC	pcDNA3-STAT5A-1*6	SalI
	Reverse	GTGGCTTCAC**GAA**TCCGTCGACTGCTTTCGCAAGTACAGG		
K696Q	Forward	CGAAAGCAGTTGACGGATACGTA**CAG**CCACAG ATCAAGC	pcDNA3-STAT5A-1*6	SnaBI
	Reverse	GCTTGATCTGTGG**CTG**TACGTATCCGTCAACTGCTTTCG		
K696R	Forward	GCGAAAGCAGTTGACGGATACGTA**CGG**CCACAGATCAAGCAAGT	pcDNA3-STAT5A-1*6	SnaBI
	Reverse	ACTTGCTTGATCTGTGG**CCG**TACGTATCCGTCAACTGCTTTCGC		
K700Q	Forward	TGAAGCCACAGATC**CAG**CAAGTGGTACCTGAGTTCGTCAA	pcDNA3-STAT5A-1*6	KpnI
	Reverse	TTGACGAACTCAGGTACCACTTG**CTG**GATCTGTGGCTTCA		
K700R	Forward	CGTGAAGCCACAGATC**CGG**CAAGTGGTACCTGAGTTCGTCAATG	pcDNA3-STAT5A-1*6	KpnI
	Reverse	CATTGACGAACTCAGGTACCACTTG**CCG**GATCTGTGGCTTCACG		
K675Q, K681Q	Forward	CAGACCGACCC**CAG**GACGAGGTCTTTGCC**CAA**TATTACACTC	pcDNA3-STAT5A-1*6 3xQ	SspI
	Reverse	GAGTGTAATA**TTG**GGCAAAGACCTCGTC**CTG**GGGTCGGTCTG		
K675R, K681R	Forward	CAGACCGACCC**CGG**GACGAGGTCTTTGCC**AGG**TATTACAC	pcDNA3-STAT5A-1*6 3xR	SmaI
	Reverse	GTGTAATA**CCT**GGCAAAGACCTCGTC**CCG**GGGTCGGTCTG		
K696Q, K700Q	Forward	GACGGATACGTA**CAG**CCACAGATC**CAG**CAAGTGGTCC	pcDNA3-STAT5A-1*6 & K689Q	SnaBI
	Reverse	GGACCACTTG**CTG**GATCTGTGG**CTG**TACGTATCCGTC		
K696R, K700R	Forward	GACGGATACGTA**AGG**CCACAGATC**AGG**CAAGTGGTCC	pcDNA3-STAT5A-1*6 & K689R	SnaBI
	Reverse	GGACCACTTG**CCT**GATCTGTGG**CCT**TACGTATCCGTC		

Control (ScI) and mouse HDAC-specific siRNA duplexes

Name		5'-3' sequence	Source	Refer to Figure
ScI	Antisense	CAGUCGCGTTTGCGACUGG[dT][dT]	Dharmacon	S4 A-G
	Sense	CCAGUCGCAAACGCGACUG[dT][dT]		
HDAC1	Antisense	GCAGCGUCUCUUUGAGAAC[dT][dT]	Dharmacon	S4 A-D, F
	Sense	GUUCUCAAAGAGACGCUGC[dT][dT]		
HDAC2	Antisense	GCAUCAGGGUUCUGCUAUG[dT][dT]	Dharmacon	S4 A-G
	Sense	CAUAGCAGAACCCUGAUGC[dT][dT]		
HDAC3*	Antisense	UUGGUAUCCUGGAGCUGCU[dT][dT]	Dharmacon	S4 A-D
	Sense	AGCAGCUCCAGGAUACCAA[dT][dT]		
HDAC3	Antisense	GUAUCCUGGAGCUGCUUAA[dT][dT]	SIGMA	S4 E-F
	Sense	UUAAGCAGCUCCAGGAUAC[dT][dT]		
HDAC4	Antisense	GAAAUUACGCUCAAGGCUU[dT][dT]	SIGMA	S4 G
	Sense	AAGCCUUGAGCGUAAUUUC[dT][dT]		
HDAC5	Antisense	GUGACACGGUGUGGAAUGA[dT][dT]	SIGMA	S4 E-F
	Sense	UCAUUCCACACCGUGUCAC[dT][dT]		
HDAC6	Antisense	GGUGCCAACUUUGACUCCA[dT][dT]	Dharmacon	S4 A-D
	Sense	UGGAGUCAAAGUUGGCACC[dT][dT]		
HDAC7	Antisense	GAACACUUUCCCUUGCGUA[dT][dT]	SIGMA	S4 G
	Sense	UACGCAAGGGAAAGUGUUC[dT][dT]		
HDAC8	Antisense	GGACGGUACUACAGUGUCA[dT][dT]	Dharmacon	S4 A-D
	Sense	UGACACUGUAGUACCGUCC[dT][dT]		
HDAC9	Antisense	CAUCCUACAAGUACACAUU[dT][dT]	SIGMA	S4 G
	Sense	AAUGUGUACUUGUAGGAUG[dT][dT]		
HDAC10	Antisense	GAAUUCUAGCUGUAGUGGA[dT][dT]	SIGMA	S4 G
	Sense	UCCACUACAGCUAGAAUUC[dT][dT]		
HDAC11.1	Antisense	GCGAGUAUACAUCAUGGAU[dT][dT]	SIGMA	S4 G
	Sense	AUCCAUGAUGUAUACUCGC[dT][dT]		
HDAC11.2	Antisense	CUAUCAAGUUCCUGUUUGA[dT][dT]	SIGMA	S4 G
	Sense	UCAAACAGGAACUUGAUAG[dT][dT]		

Quantitative RT-PCR primers for mouse gene expression analysis (Source: Metabion, SIGMA) - Amplicon length : 51 - 254 bp

Gene name		5'-3' sequence	Type of gene	Amplicon location
S9	Forward	GCAAGATGAAGCTGGATTAC	Housekeeping gene (normalizer)	Coding sequence (CDS)
	Reverse	GGGATGTTCACCACCTG		
36b4	Forward	GCGTCCTGGCATTGTCTGT	Housekeeping gene (normalizer)	CDS
	Reverse	GCCGCAAATGCAGATGG		
Cis	Forward	CTGGACTCTAACTGCTTGTC	STAT5 target gene	CDS
	Reverse	TAGGCAGCACCGAGTCAC		
c-Myc	Forward	AACAGGAACTATGACCTCG	STAT5 target gene	CDS
	Reverse	AGCAGCTCGAATTTCTTC		
Osm	Forward	AGATACCTGAGCCCACACAGACAG	STAT5 target gene	CDS
	Reverse	ATCGTCCCATTCCCTGAAGACC		
c-Fos	Forward	CGAAGGGAACGGAATAAGATGG	Control gene	CDS
	Reverse	AGACCTCCAGTCAAATCCAGGG		
p21	Forward	TGATGTCCGACCTGTTCCG	Control gene	CDS
	Reverse	CCGAAGAGACAACGGCACA		
Brd2	Forward	GAGAGCCCCACAATGGCTT	BET family gene	CDS
	Reverse	TGGTTTGTTACCCGTCCTGG		
Hsp70	Forward	TGCTGGACAAGTGCCAGGA	Control gene	CDS
	Reverse	TGGTACAGCCCACTGATGATG		
HDAC1	Forward	AACAGAGGATGAGAAAGAGAAAGATC	HDAC family gene	CDS
	Reverse	TCAGGCCAACTTGACCTCTTC		
HDAC2	Forward	AAGAAGACAAGAAGGAGACAGAGG	HDAC family gene	CDS
	Reverse	TCAAGGGTTGCTGAGTTGTTC		
HDAC3	Forward	TCCCGGCAGACCTCCTGACG	HDAC family gene	CDS
	Reverse	TTTCCTTGTCGTTGTCATGGTCGC		
HDAC4	Forward	CACTCCTGCGTCCTGCCTT	HDAC family gene	3' UTR
	Reverse	CGCCTGCTGTCCCTTTGTAC		
HDAC5	Forward	ACGCCTCCCTCCTACAAATTG	HDAC family gene	CDS
	Reverse	GGAAAGTCATCACGGCTGTCA		
HDAC6	Forward	GGCCAAGATTCTTCTACTAGACAGCG	HDAC family gene	CDS
	Reverse	CTAGATTGGGGCTGGAGTGGG		
HDAC7	Forward	GCCCTGCCCTCCAGCCAGAC	HDAC family gene	CDS
	Reverse	GGTGTTGCAGGGTCAGCAGCG		
HDAC8	Forward	CTGCGACTCCCTTGTGAAGG	HDAC family gene	CDS
	Reverse	GCAGGGCATAGGCTTCGAT		
HDAC9	Forward	AGTTCACCAAACAATGGCCC	HDAC family gene	CDS
	Reverse	TGAAGCCTCATTTTCGGTCAC		
HDAC10	Forward	GCCTGTGTTTGTCAGCTTGTG	HDAC family gene	CDS
	Reverse	GGGCTATATATTCCGGGCTGT		
HDAC11	Forward	GGTGACCTCGGGTGGGTAC	HDAC family gene	CDS
	Reverse	GAGACGCAGGGAAACTCAGG		

Chromatin immunoprecipitation quantitative PCR primers specific for mouse genomic DNA (Source: Metabion, SIGMA) - Amplicon length : 52 - 85 bp

Name & position[1]		5'-3' sequence	Gene	Amplicon location
Cis -831/-755	Forward	AGGGCTGTCTGGGAGCTGA	STAT5 target gene Cis	Distal promoter
	Reverse	TCTCTGAGTGGACCGACAGTTG		
Cis -259/-199	Forward	CAACTCTAGGAGCTCCCGCC	STAT5 target gene Cis	STAT5 binding sites (1+2)
	Reverse	AACACCTTTGACAGATTTCCAAGAAC		
Cis -188/-104	Forward	GTCCAAAGCACTAGACGCCTG	STAT5 target gene Cis	STAT5 binding sites (3+4)
	Reverse	TTCCCGGAAGCCTCATCTT		
Cis -18/+55	Forward	GTTCGCACCACAGCCTTTCAGTCC	STAT5 target gene Cis	Transcription start site (TSS)
	Reverse	GTCCAGGGGTGCGAAGGTCAGG		
Cis +261/+322	Forward	GGACTTCGAGTGGTGTGCCTA	STAT5 target gene Cis	ORF (beginning)
	Reverse	GGCTCCGTTTCCCTATCCA		
Cis +502/+553	Forward	CATTCCTCCGTCCCAGGTC	STAT5 target gene Cis	ORF
	Reverse	ACCTCAGGCTGGCTTCCTAAG		
Cis +1061/+1112	Forward	AATTTTCGGACTCTTCGGCA	STAT5 target gene Cis	ORF
	Reverse	CACCCAAGAAAGGAAGGCAG		
Cis +2236/+2308	Forward	GAGGACACTGCCTTCCCTCA	STAT5 target gene Cis	ORF
	Reverse	AAGCTTCTACCCACTCCGGC		
Cis +3963/+4029	Forward	TACCCCTTCCAACTCTGACTGAGC	STAT5 target gene Cis	ORF (end)
	Reverse	TTCCCTCCAGGATGTGACTGTG		
Osm -184/-122	Forward	CATCATCCTTGGGCGTGGGGC	STAT5 target gene Osm	STAT5 binding sites (1+2)
	Reverse	CGCTCCTCCTCCCGTTTTCTTCG		
Osm +25/+87	Forward	GCTGCCAGCCTGCAGGACAC	STAT5 target gene Osm	TSS
	Reverse	GTACTCTGGCCCGTGCCTCTCAG		
c-Fos -259/-200	Forward	GACCATCTCCGAAATCCTACACGC	Control gene c-Fos	Proximal promoter
	Reverse	CACATTTGGGATCTTAGGGGGTCTC		
c-Fos -70/-1	Forward	GGAAGTCCATCCATTCACAGCG	Control gene c-Fos	TSS
	Reverse	CAGTCGCGGTTGGAGTAGTAGGC		
p21 -120/-61	Forward	GAGGGCGGGCCAGCGAGTC	Control gene p21	Proximal promoter
	Reverse	CTCAGAGGCAGGACCAACCCACTC		
p21 +75/+136	Forward	ATCCAGACATTCAGAGGTGAGAGC	Control gene p21	TSS
	Reverse	CATTGCTACGGGGAAGAACTATTG		

[1]Position relative to the transcription start site (TSS)

Table S2. Review of reported IC_{50} (±SD) of *in vitro* inhibition of recombinant human HDACs by pan- and class I-selective deacetylase inhibitors

	class I				class II A				class II B		class IV		
	HD1	HD2	HD3	HD8	HD4	HD5	HD7	HD9	HD6	HD10	HD11	References	>50% Inhibition of STAT5 activity[1]
**pan-inhibitors**													
**TSA (nM)**	**3.45 (±3.61)**	**2.00 (±1.15)**	**2.06 (±1.61)**	319.13 (±157.79)	**7.84 (±2.60)**	260.00 (*)	400.00 (±528.23)	403.00 (±561.44)	**5.90 (±5.91)**	**10.75 (±1.06)**	**31.00 (*)**	(2–12)	**20 nM**
**SAHA (nM)**	**63.62 (±43.33)**	**99.38 (±74.84)**	**63.10 (±41.75)**	**796.33 (±576.30)**	**501.25 (±732.90)**	1600.33 (±1802.36)	1130.67 (±1225.92)	**856.75 (±1496.66)**	**48.22 (±47.21)**	**89.67 (±57.06)**	3093.33 (±4859.23)	(2–7, 12–22)	**1000 nM[2]**
**class I inhibitors**													
**Valproic Acid (μM)**	**774.33 (±775.18)**	**467.33 (±374.33)**	**1410.67 (±1497.84)**	**3772.50 (±5189.46)**	**1750.00 (±353.55)**	**1500.00 (±707.11)**	**1650.00 (±494.97)**	**2000.00 (*)**	**8133.33 (±10278.78)**	20000.00 (*)	ND	(3, 12, 23, 24)	**2000 μM**
**Apicidin (nM)**	**11.93 (±21.39)**	**40.67 (±68.70)**	**11.33 (±21.12)**	**481.00 (±407.26)**	10000.00 (*)	ND	10000.00 (*)	10000.00 (*)	9800.00 (±282.84)	ND	ND	(3, 4, 7, 12, 22)	**100 nM**
**MGCD0103 (nM)**	**33.50 (±34.41)**	**34.67 (±1.15)**	**310.75 (±210.38)**	15000.00 (±8660.25)	10000.00 (±0.00)	10000.00 (*)	10000.00 (±0.00)	10000.00 (*)	16666.67 (±11547.01)	5799.00 (*)	**392.50 (±279.31)**	(3, 12, 13, 21, 25, 26)	**None (2500 nM)**
**MS-275 (nM)**	**171.38 (±121.34)**	**411.67 (±644.23)**	**384.40 (±247.08)**	62075.00 (±27217.32)	10350.00 (±494.97)	ND	10000.00 (*)	**505.00 (*)**	55750.00 (±51110.18)	50100.00 (*)	ND	(2–4, 7, 9, 10, 12, 21, 22)	**None (20000 nM)**

Legend: **strong inhibition** **weak inhibition** no inhibition ND, not determined (*) one report only [1]this study [2]previous study (27)

Ba/F3-1*6

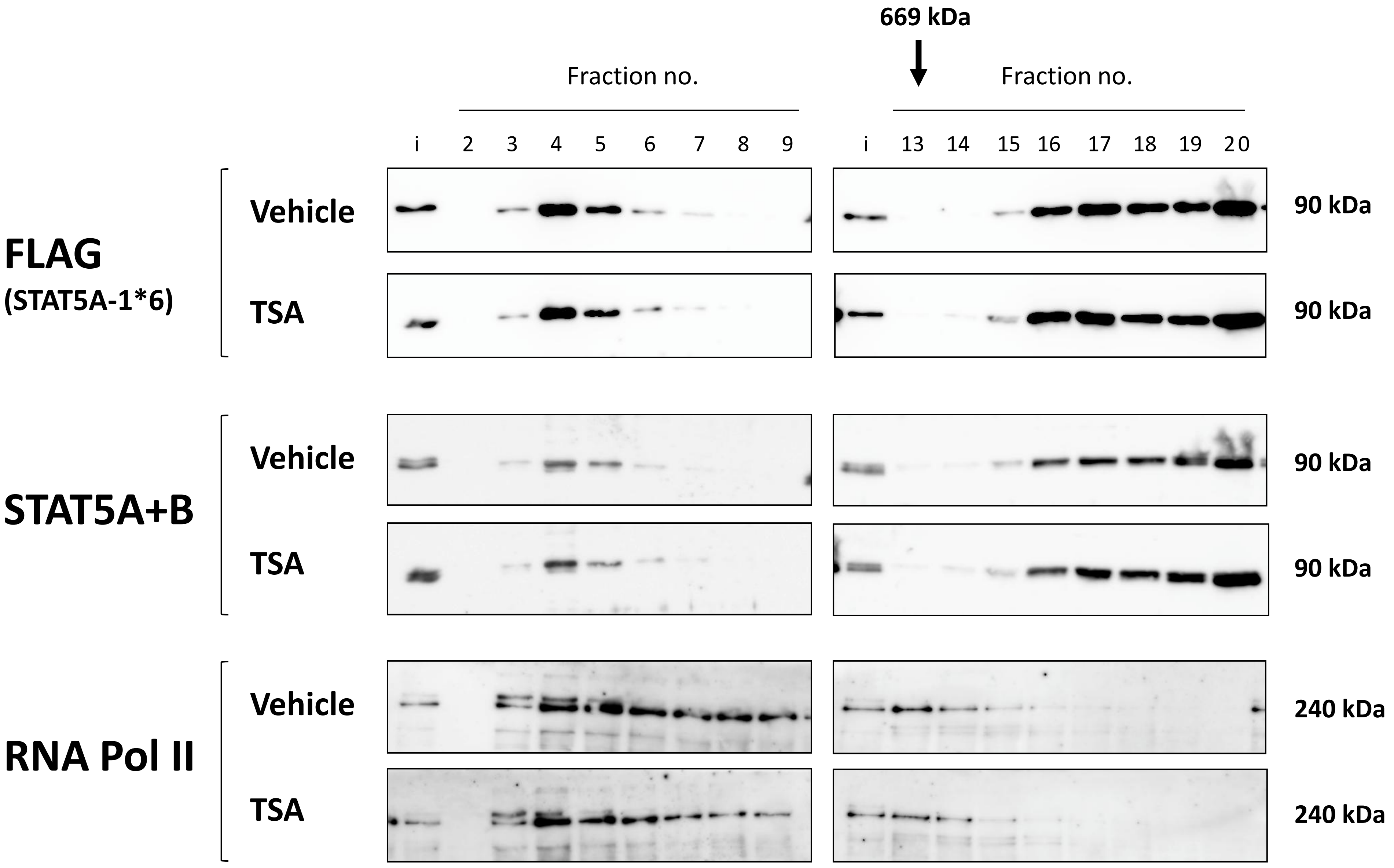

Figure S1 (Pinz et al.)

Figure S2 (Pinz et al.)

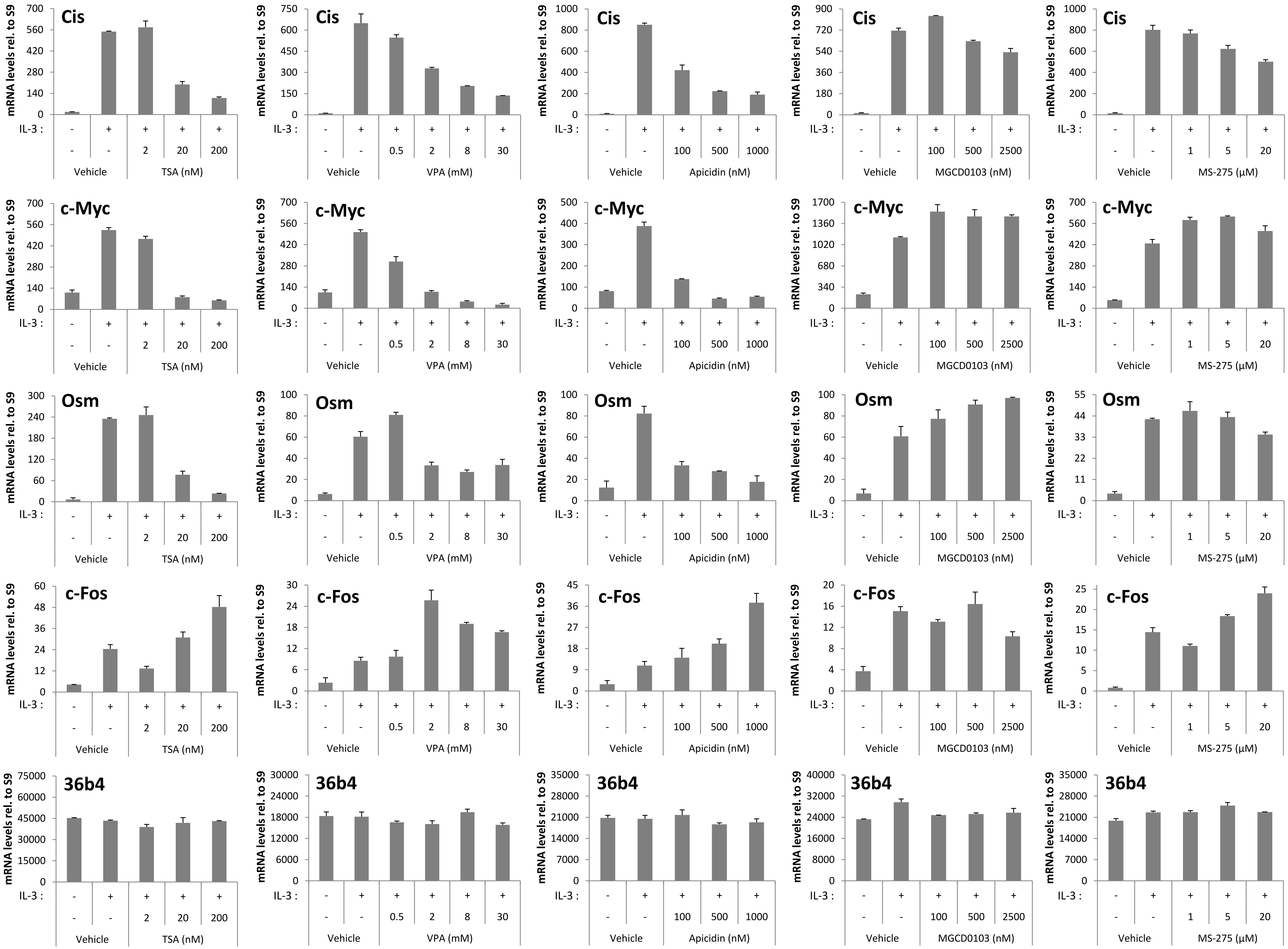

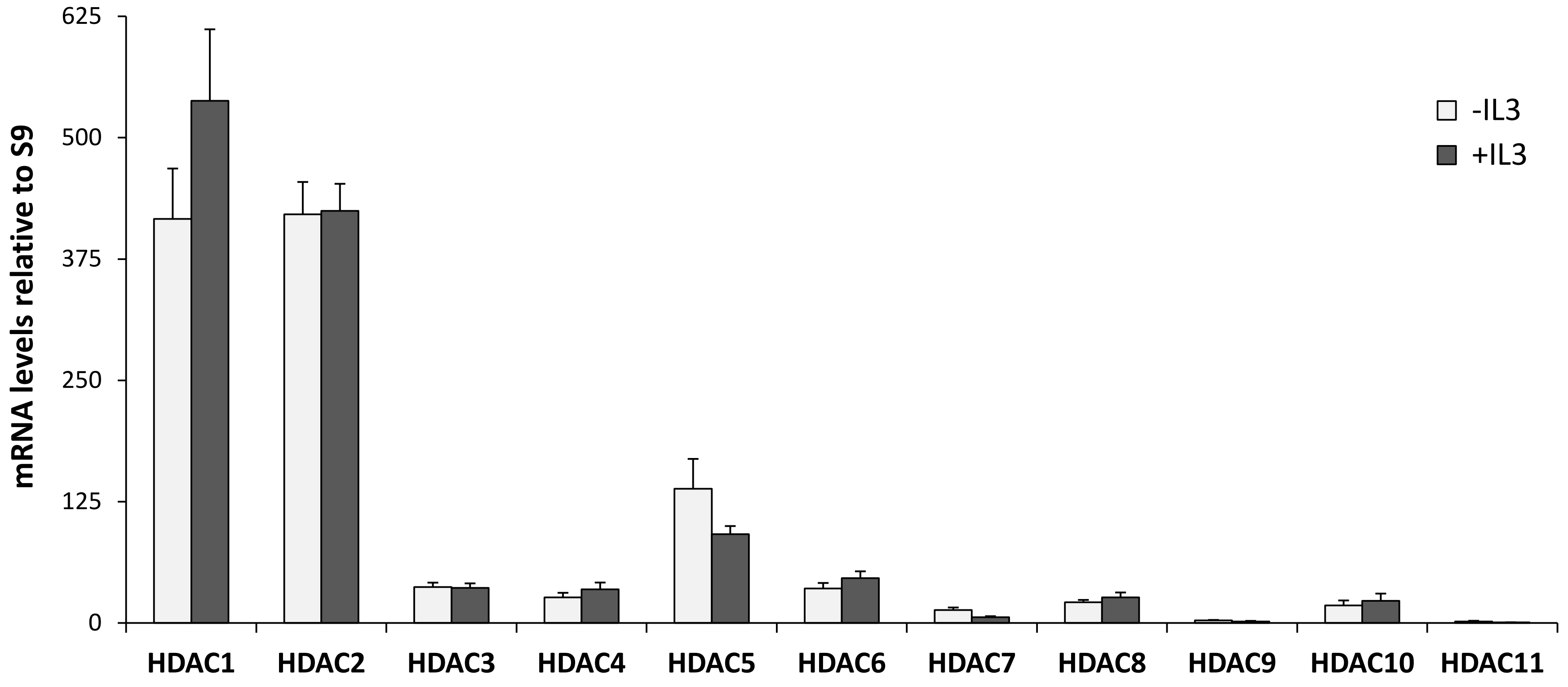

Figure S3 (Pinz et al.)

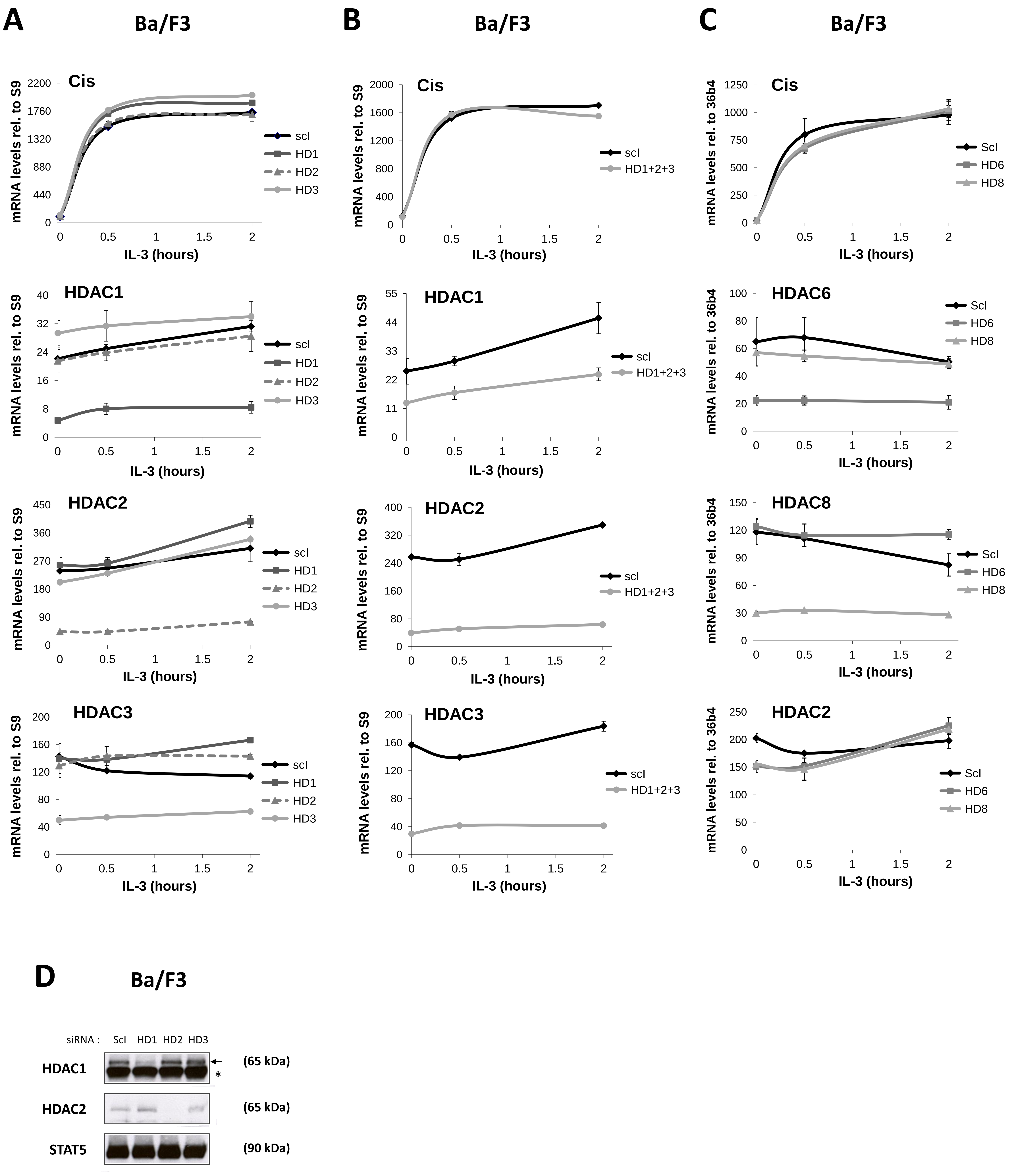

Figure S4, A-D (Pinz et al.)

E

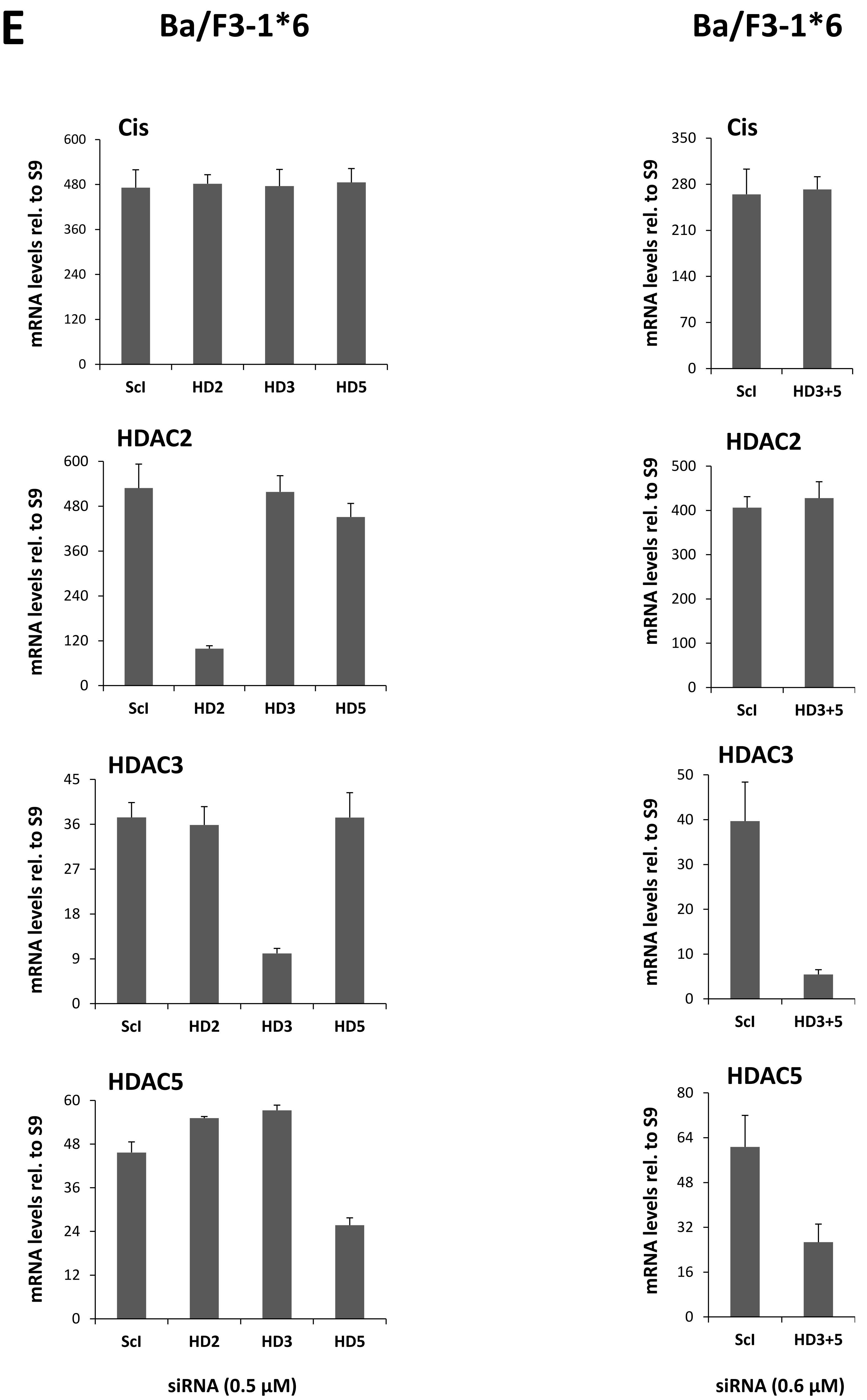

F

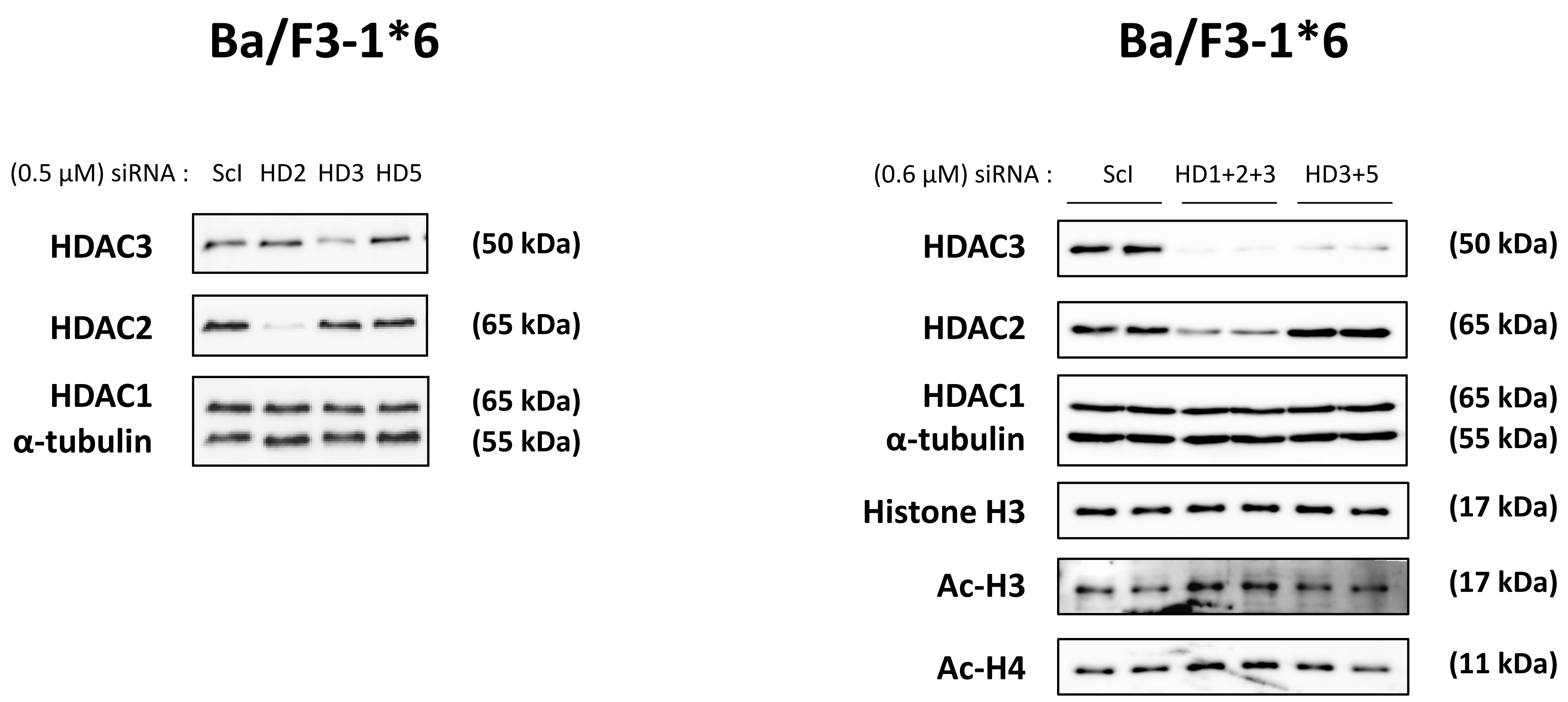

Figure S4, E-F (Pinz et al.)

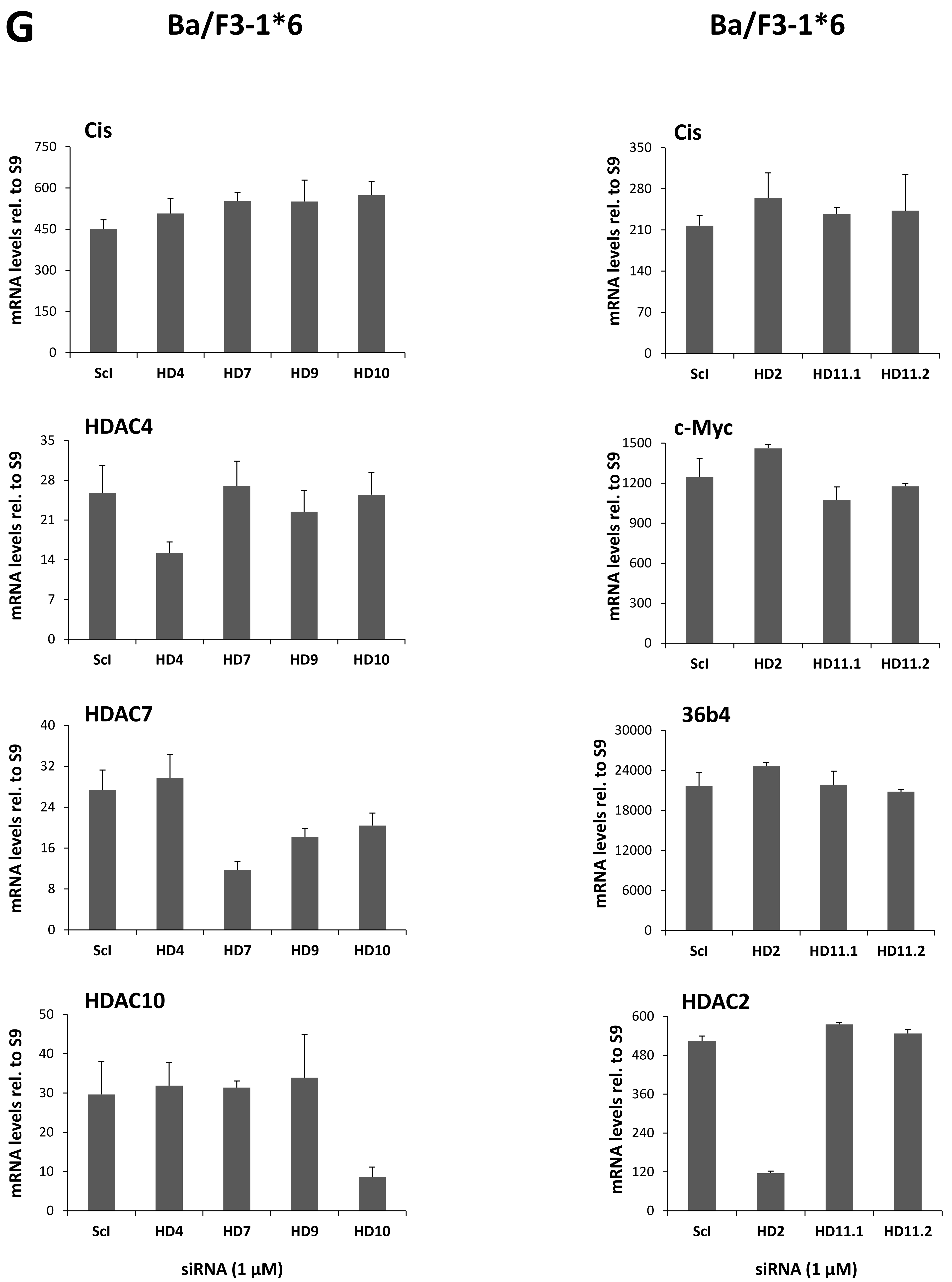

Figure S4, G (Pinz et al.)

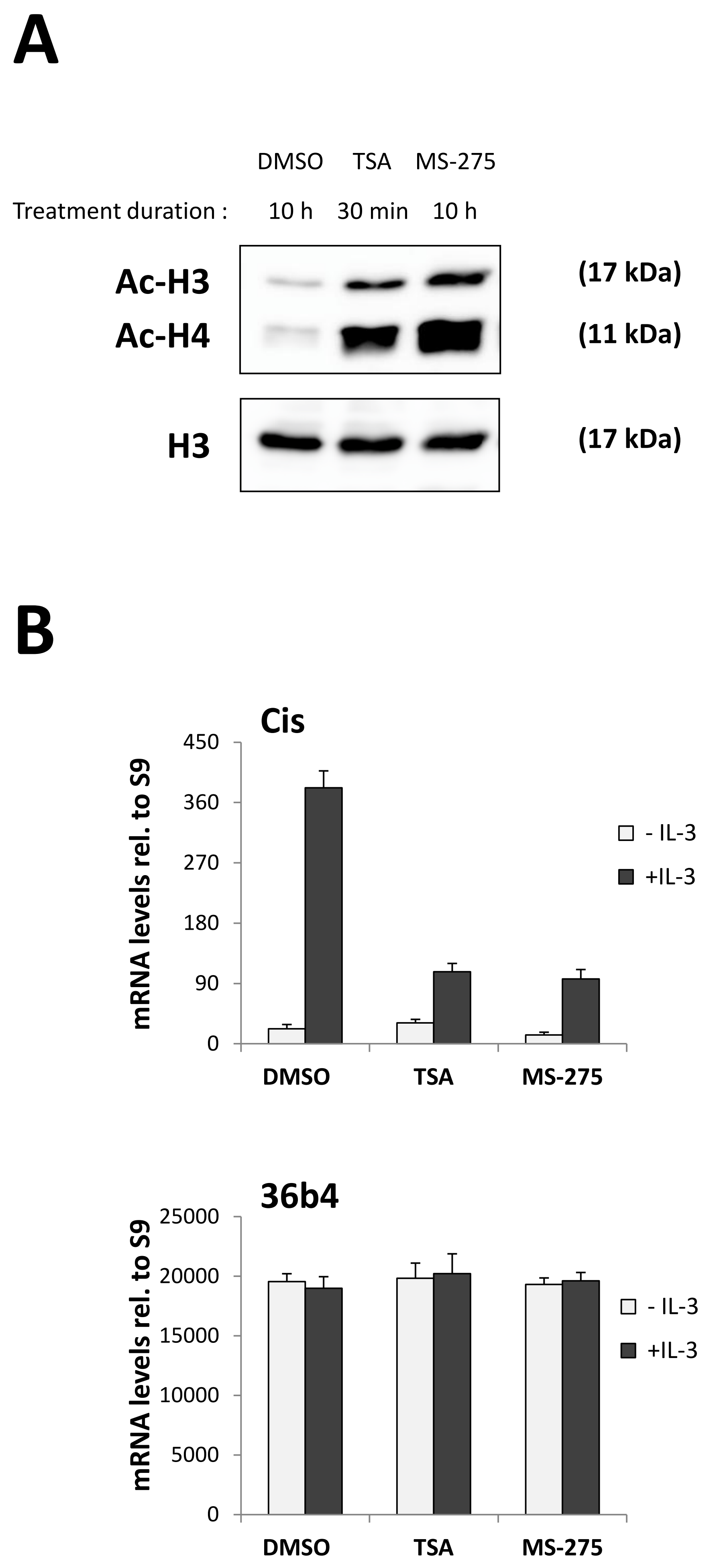

Figure S5 (Pinz et al.)

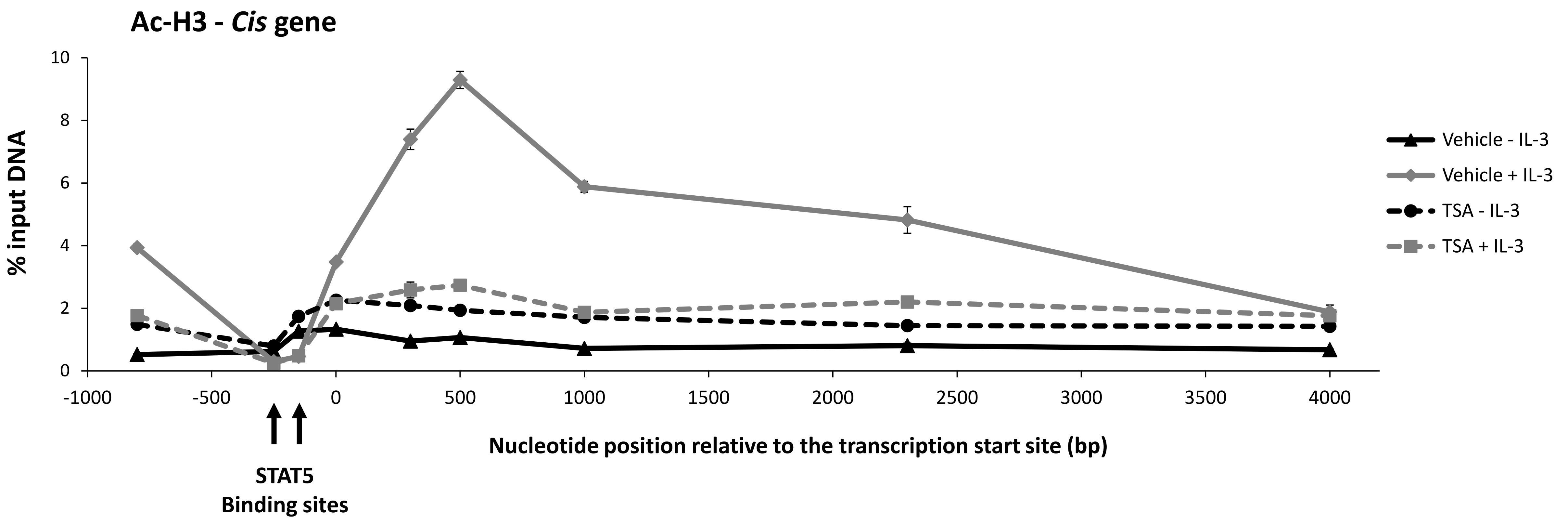

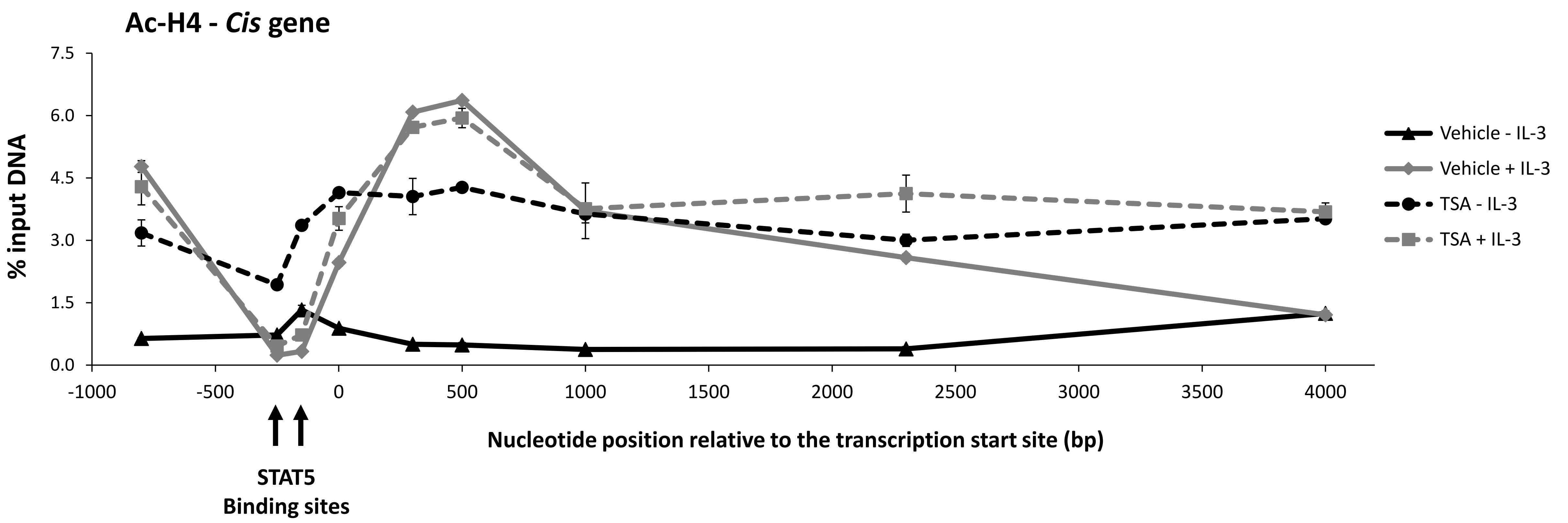

Figure S6 (Pinz et al.)

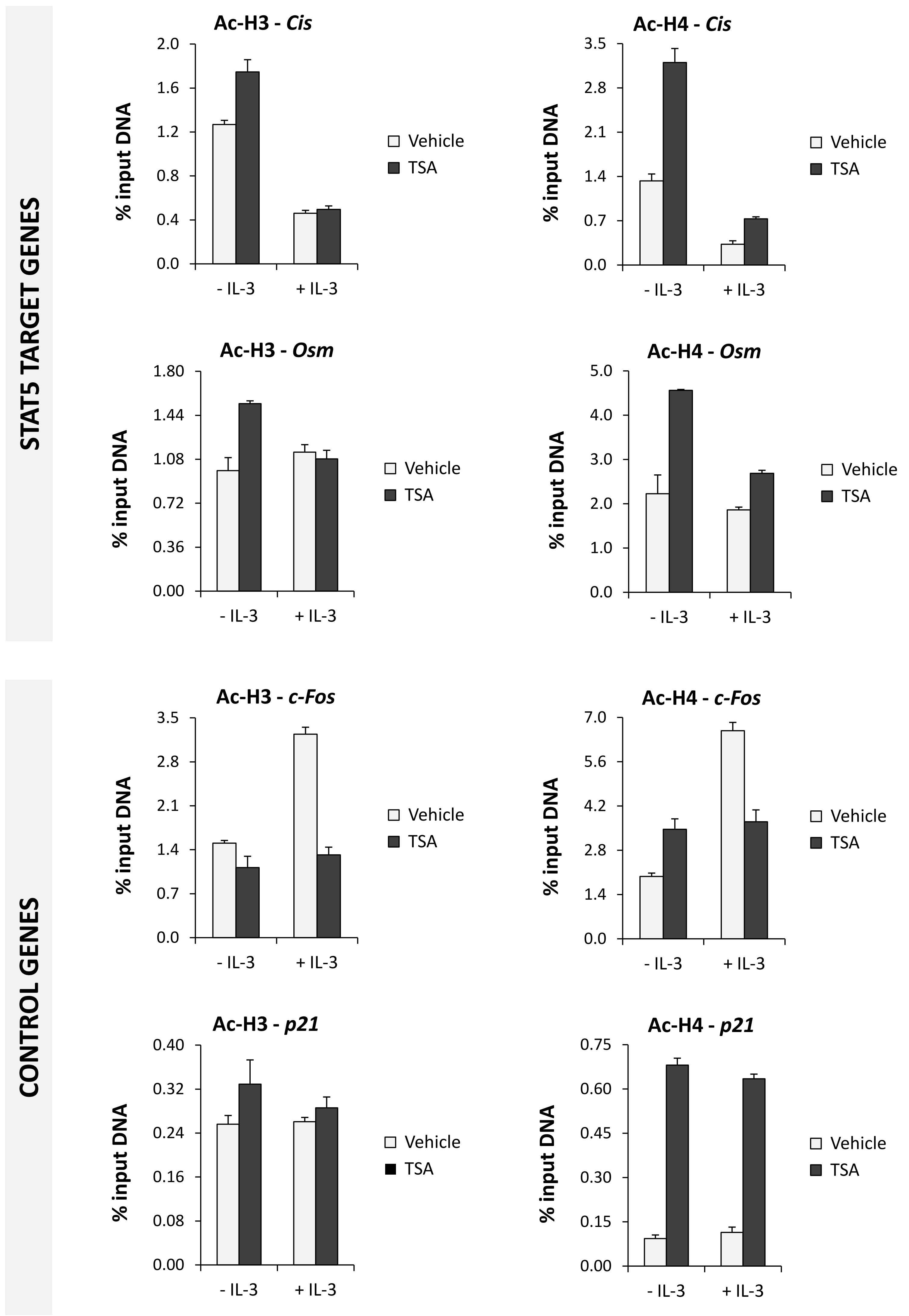

Figure S7 (Pinz et al.)

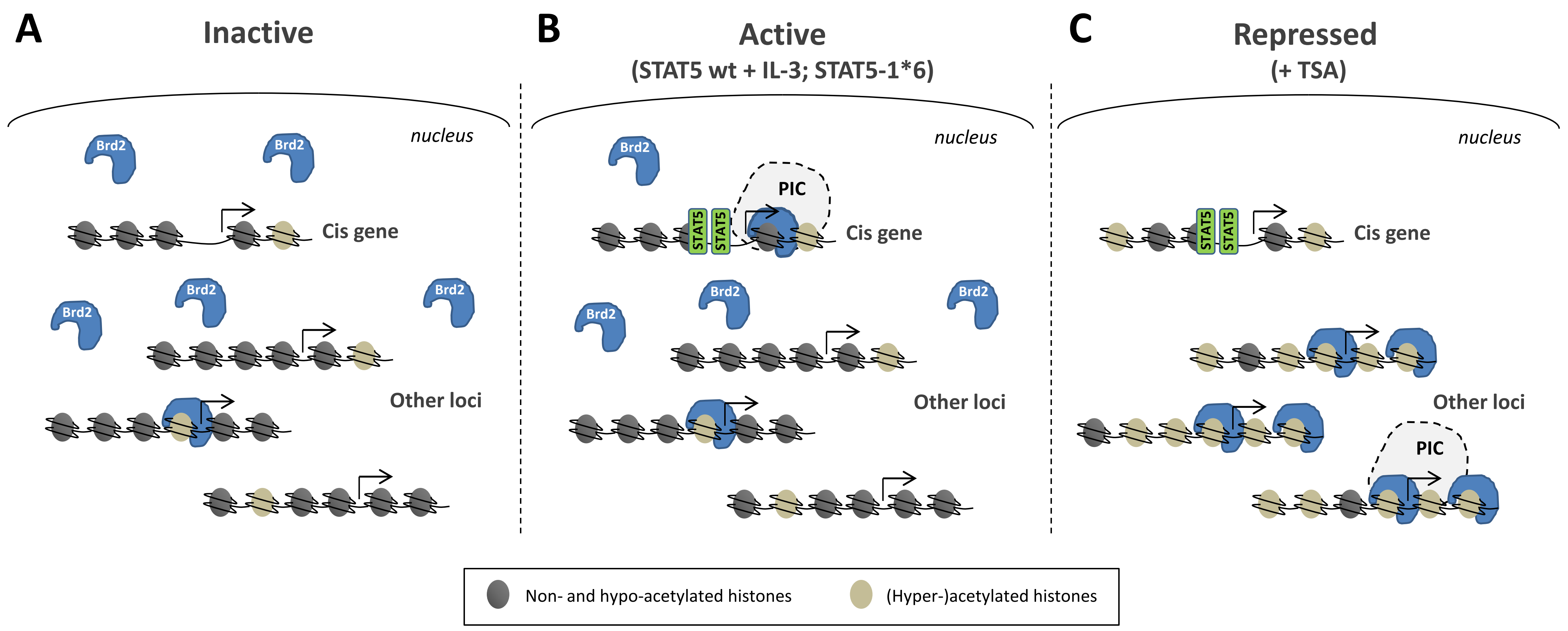

Figure S8 (Pinz et al.)
